# New taxa, including three new genera show uniqueness of Neotropical Nepticulidae (Lepidoptera)

**DOI:** 10.3897/zookeys.628.9805

**Published:** 2016-10-31

**Authors:** Erik J. van Nieukerken, Camiel Doorenweerd, Kenji Nishida, Chris Snyers

**Affiliations:** 1 Naturalis Biodiversity Center, PO Box 9517, NL-2300 RA Leiden, The Netherlands; 2 Estación Biológica Monteverde, Apdo. 22–5655, Monteverde, Costa Rica; 3 Rendierstraat 14/2, BE-2610 Wilrijk, Antwerpen, Belgium

**Keywords:** New species, new genus, taxonomy, leafmines, gall, molecular phylogeny

## Abstract

After finding distinct clades in a molecular phylogeny for Nepticulidae that could not be placed in any known genera and discovering clear apomorphic characters that define these clades, as well as a number of Neotropical species that could be placed in known genera but were undescribed, three new genera and nine new species are here described from the Neotropics: *Stigmella gallicola* van Nieukerken & Nishida, **sp. n.** reared from galls on *Hampea appendiculata* (Malvaceae) in Costa Rica, representing the first example of a gall making *Stigmella*; *Stigmella schinivora* van Nieukerken, **sp. n.** reared from leafmines on *Schinus terebinthifolia* (Anacardiaceae) in Argentina, Misiones; *Stigmella costaricensis* van Nieukerken & Nishida, **sp. n.** and *Stigmella intronia* van Nieukerken & Nishida, **sp. n.** each from a single specimen collected the same night in Costa Rica, Parque Nacional Chirripó; *Stigmella molinensis* van Nieukerken & Snyers, **sp. n.** reared from leafmines on *Salix humboldtiana*, Peru, Lima, the first Neotropical species of the *Stigmella salicis* group sensu stricto; *Ozadelpha* van Nieukerken, **gen. n.** with type species *Ozadelpha conostegiae* van Nieukerken & Nishida, **sp. n.**, reared from leafmines on *Conostegia oerstediana* (Melastomataceae) from Costa Rica; *Neotrifurcula* van Nieukerken, **gen. n.** with type species *Neotrifurcula gielisorum* van Nieukerken, **sp. n.** from Chile; *Hesperolyra* van Nieukerken, **gen. n.**. with type species *Fomoria diskusi* Puplesis & Robinson, 2000; *Hesperolyra saopaulensis* van Nieukerken, **sp. n.**, reared from an unidentified Myrtaceae, Sao Paulo, Brasil; and *Acalyptris janzeni* van Nieukerken & Nishida, **sp. n.** from Costa Rica, Guanacaste. Five new combinations are made: *Ozadelpha ovata* (Puplesis & Robinson, 2000), **comb. n.** and *Ozadelpha guajavae* (Puplesis & Diškus, 2002), **comb. n.**, *Hesperolyra diskusi* (Puplesis & Robinson, 2000), **comb. n.**, *Hesperolyra molybditis* (Zeller, 1877), **comb. n.** and *Hesperolyra repanda* (Puplesis & Diškus, 2002), **comb. n.** Three specimens are briefly described, but left unnamed: *Ozadelpha* specimen EvN4680, *Neotrifurcula* specimen EvN4504 and *Neotrifurcula* specimen RH2.

## Introduction

The nature of Latin America is generally known to be both very diverse and peculiar in its composition and Lepidoptera are no exception to this phenomenon. Biogeographically the region is usually known as the Neotropical region, or the Neotropics, although especially botanists often exclude the southern – non tropical – part (Antonelli and Sanmartín 2011; Morrone 2014). Probably more than one third of the globally named Lepidoptera species can be found in the Neotropics (Heppner 1991), although the number given by Heppner very likely is still a huge underestimate (Kristensen et al. 2007). The peculiarity is evident from many of the phylogenetically lower Lepidoptera, the non-Ditrysia, for example the endemic jaw moth family Heterobathmiidae in Patagonia, the endemic Andesianidae, the Palaephatidae, shared only with Australia, amongst which the supposed sister group to the Ditrysia is to be found and other rare southern families such as Cecidosidae and Neopseustidae (Nielsen 1985; Regier et al. 2015; Bazinet et al. 2016). Most of the families with typically leafmining larvae also include endemic New World or Neotropical groups or radiations: in Heliozelidae the basal Patagonian genus *Plesiozela* Karsholt & Kristensen, 2003 (Karsholt and Kristensen 2003), in Tischeriidae a radiation of the New World endemic *Astrotischeria* Puplesis & Diškus, 2003, specialised on Asteraceae (Diškus and Puplesis 2003) and in Opostegidae the endemic genera *Notiopostega* Davis, 1989 and *Neopostega* Davis & Stonis, 2007 (Davis 1989; Davis and Stonis 2007). Only the leafmining family Nepticulidae did not have endemic Neotropical genera.

Until fairly recently the nepticulid fauna of the Neotropics was almost completely unstudied, and twelve of the species described between 1877 and 1962 were tentatively placed in the large global genus *Stigmella* Schrank, 1802, with just a single species in the – at the time – monotypic genus *Enteucha* Meyrick, 1915 (Davis 1984). Collecting and rearing leafmines was rarely done in Latin America, with the notable exception of Bourquin (1961) who described the first two South American Asteraceae feeding nepticulids. During their expeditions to Patagonia in the 1970’s and 80’s both the Danish researchers Ebbe Nielsen and Ole Karsholt and Don Davis from the USA collected Nepticulidae, mostly adults, and other non-Ditrysian moth families (Nielsen 1985), but this material remained unstudied for some time. A research team led by the Lithuanian researcher Rimantas Puplesis (later named Jonas R. Stonis) started fieldwork in Latin America in the late 1990’s and published a revision of Neotropical nepticulids early this century, bringing the total to 74 species in seven genera by 2002 (Puplesis and Robinson 2000; Puplesis et al. 2002a, 2002b), including several species only known from southern Florida. Since then they continued with papers describing 40 new species, bringing the total to 114 in July 2016 (Šimkevičiūtė et al. 2009; Stonis et al. 2013a, 2013b, 2013c, 2014, 2015, 2016; Diškus and Stonis 2014; Remeikis et al. 2014; Remeikis and Stonis 2015; Stonis and Remeikis 2015, 2016). These revisions continue, fieldwork continues and collections in Copenhagen, Washington and Vilnius still contain unnamed material (J. R. Stonis personal communication).

Even though almost all Neotropical species up to now have been placed in known nepticulid genera, which have been described from other parts of the world, several species show unique combinations of characters, and their placement is therefore disputable. Only the genera *Enteucha* Meyrick, 1915 and *Manoneura* Davis, 1979 were described originally from the Neotropics (respectively British Guyana and southern Florida), but *Enteucha* is not confined to the Neotropics, and also occurs in the Palearctic and Oriental regions (van Nieukerken 1986; van Nieukerken et al. 2016). The monotypic *Manoneura* was synonymised with *Enteucha* by van Nieukerken (1986). Puplesis and Robinson (2000) reinstated *Manoneura* as a good genus on the basis of autapomorphic characters, and described a second species, but it was synonymised again after it was placed with high confidence inside a monophyletic *Enteucha* clade in a molecular phylogeny with eight genes (Doorenweerd et al. 2016; van Nieukerken et al. 2016). Peculiar taxa include the species placed in *Glaucolepis* Braun, 1917, *Fomoria* Beirne, 1945 and several in *Enteucha*; also some species in *Acalyptris* Meyrick, 1921, particularly the *Acalyptris latipennata* group, show combinations of characters unknown in this genus elsewhere. Also the single Neotropical species in *Ectoedemia* Busck, 1907 (*Ectoedemia fuscivittata* Puplesis & Robinson, 2000) has an aberrant morphology.

While working on the molecular phylogeny of global Nepticulidae, to be published almost simultaneously (Doorenweerd et al. 2016), the sampling initially lacked Neotropical material and EvN and CD thus attempted to obtain fresh material for DNA analysis through several sources. This comprised very limited collecting by EvN in 2000 and some material received from others, further KN had been actively collecting and rearing nepticulids in Costa Rica the last decades, and CS collected leafmines during his annual visits to Lima since 2010.

Even though we could still only include a limited set of material compared to the described diversity, the analyses based on DNA sequences yielded several surprises. Three clades with distinctly long branches in the phylogeny could not be placed in any known genus, and other Neotropical species that were studied and could be placed in known genera also appeared to be hitherto unnamed. Taxonomists working on Nepticulidae fortunately have the tradition to be reluctant in describing new genera, preventing the systematic chaos that has been evident in various other groups of Lepidoptera. It is only after careful consideration of the morphological and molecular evidence that we here name these genera as well as several species, of which the common denominators are Neotropics and availability of DNA sequences (minimally a DNA barcode), and often provide additional interesting information on biology.

We describe three new genera and nine new species, including the first nepticulid species from Brazil and Costa Rica and the first known gall-forming *Stigmella* species.

## Material and methods

### Material

Material was either collected by one of the authors or loaned from various institutions. The Costa Rica material was collected by Kenji Nishida, and made available as loan from the Museo de Zoología, Escuela de Biología, Universidad de Costa Rica
(MZUCR), allowing for further study, including DNA analysis. Some of the specimens, including all holotypes were later donated to Naturalis (RMNH), because of its large collection of global Nepticulidae. Holotypes of the species from Brazil, collected by Erik van Nieukerken and Peru, collected by Chris Snyers are deposited respectively in DZUP and UNALM. On the basis of DNA barcodes, analysed by us, we could match several specimens from the global Malaise trapping program to three of our new species, for two of these we borrowed some material from BIOUG, stored in ethanol 96%, to check the genitalia. We also cite the other specimens from the Malaise trappings with matching DNA barcodes that we did not study. The Argentinean samples from this program are returned to MACN and the Costa Rican samples are donated to RMNH. We select a Holotype of each species, but refrain from designating and labelling paratypes, as these have no name-bearing function in Zoological Nomenclature (International Commission on Zoological Nomenclature 1999). All material is also listed in Suppl. material 1.

**Abbreviations for depositories etc.**

BIN
 Barcode Index Number  (Ratnasingham and Hebert 2013)

BIOUG
Biodiversity Institute of Ontario, University of Guelph, Canada 


BMNH
Natural History Museum, London, UK 


BOLD
 Barcode of Life data Systems  (http://www.barcodinglife.com/)

DZUP
Universidade Federal do Parana, Coleção de Entomológica Padre Jesus Santiago Moure, Brazil 


MACN
Museo Argentino de Ciencias Naturales, Bernardino Rivadavia, Buenos Aires, Argentina 


MZUCR
 Museo de Zoología, Universidad de Costa Rica, San Pedro de Montes de Oca, San José, Costa Rica 


RMNH
Naturalis Biodiversity Center, Zoological collections, Leiden, The Netherlands 


UNALM
 Museo de Entomología “Klaus Raven Büller”, Universidad Agraria La Molina, Lima, Peru 


ZMUC
Natural History Museum of Denmark, Zoological Museum, Copenhagen, Denmark 


### Methods

Collecting and rearing methods varied depending on the collector, but generally followed commonly used methods for the collection of leaf miners.

Genitalia were prepared according to our standard procedures, usually including DNA extraction, and described earlier in detail (van Nieukerken 1985; van Nieukerken et al. 2010). Wings were denuded in ethanol 70% and stained with phenosafranin before embedding in euparal.

*Measurements*. Measurements of genitalia were obtained from digital images, using calibrated scaling in the Zeiss AxioVision software, see below, 20× objective for male genitalia and 10× or 20× for females. Capsule length was measured from vinculum to middle of uncus; valva length from tip of posterior process to ventral edge, excluding the sublateral process; phallus length was measured from the sclerotized tube, from tip of ventral process/carina, excluding any protruding vesica parts. Total bursa length includes all of the internal genitalia from cloaca to anterior edge of bursa. Genitalia measurements are usually rounded off to nearest 5µm. Forewing length was measured from tip of fringe to attachment on thorax, usually at magnification of 20×, also preferably on photographs. Antennal segment counts include scape and pedicel; they were counted on photographs or directly using the stereo microscope.

*Morphological terms*. The terminology used largely follows our earlier treatments and other recent papers on Nepticulidae (van Nieukerken 1986; van Nieukerken et al. 1990), but is slightly adapted to follow Kristensen’s (2003) detailed morphological treatment. We thus use phallus rather than aedeagus for the male intromittent organ, and adopt Wootton’s (1979) venation nomenclature, meaning that R2–5 become Rs1–4 and Cu changes into CuA. See also Doorenweerd et al. (2016).

*Photographs* of mounted moths were made with an AxioCam digital camera attached to a motorized Zeiss SteREO Discovery V12, using the Module Extended Focus, Zeiss AxioVision software, to prepare a picture in full focus from a Z-stack of 10 to 35 individual photos. Leafmines were photographed by EvN with a similar camera on a Zeiss Stemi SV11 stereo-microscope, without extended focus; other illustrated leafmines are from field photographs. Genitalia and wing slides were photographed with a similar camera on a manually operated Zeiss Axioskop H, usually with just a single exposure. Photographs were edited with Adobe Photoshop, avoiding any change to the real object, but backgrounds are cleaned from excess debris and artefacts by using healing brush and clone tools; tone and contrast are adjusted and a little sharpening is used in some photos. Photographs of venation, some mines and some female genitalia were taken in sections, and combined with the photomerge tool in Photoshop.

The photographs in this paper were taken by: Figs 17–33 by Kenji Nishida, Figs 58–65 by Chris Snyers, Figs 7 and 8 by Els Baalbergen, Figs 68, 97, 99, 117, 120 by Camiel Doorenweerd, Figs 119, 142, 143 by Cees van den Berg, Figs 67 and 73 by Jonas R. Stonis and the remaining ones by Erik van Nieukerken.

*Molecular methods* were described in detail in previous studies (Doorenweerd et al. 2015; Doorenweerd et al. 2016). The DNA barcode data and GENBANK numbers are given both in Suppl. material 1 and in BOLD dataset DS-NEONEP (doi: 10.5883/DS-NEONEP).

## Taxonomy

### Checklist

*Stigmella* Schrank, 1802

*Stigmella gallicola* van Nieukerken & Nishida, **sp. n.**

*Stigmella prunifoliella* group

*Stigmella schinivora* van Nieukerken, **sp. n.**

*Stigmella costaricensis* van Nieukerken & Nishida, **sp. n.**

*Stigmella intronia* van Nieukerken & Nishida, **sp. n.**

*Stigmella salicis* group

*Stigmella molinensis* van Nieukerken & Snyers, **sp. n.**

*Ozadelpha* van Nieukerken, **gen. n.**

*Ozadelpha conostegiae* van Nieukerken & Nishida, **sp. n.**

*Ozadelpha guajavae* (Puplesis & Diškus, 2002), **comb. n.**

*Ozadelpha ovata* (Puplesis & Robinson, 2000), **comb. n.**

*Ozadelpha* specimen EvN4680

*Neotrifurcula* van Nieukerken, **gen. n.**

*Neotrifurcula gielisorum* van Nieukerken, **sp. n.**

*Neotrifurcula* specimen EvN4504

*Neotrifurcula* specimen RH2

*Hesperolyra* van Nieukerken, **gen. n.**

*Hesperolyra diskusi* (Puplesis & Robinson, 2000), **comb. n.**

*Hesperolyra molybditis* (Zeller, 1877), **comb.n.**

*Hesperolyra repanda* (Puplesis & Diškus, 2002), **comb.n.**

*Hesperolyra saopaulensis* van Nieukerken, **sp. n.**

*Hesperolyra* species 29122 (Puplesis and Robinson 2000)

*Acalyptris* Meyrick, 1921

*Acalyptris janzeni* van Nieukerken & Nishida, **sp. n.**

### Genus *Stigmella* Schrank, 1802

#### Stigmella gallicola

Taxon classificationAnimaliaLepidopteraNepticulidae

van Nieukerken & Nishida sp. n.

http://zoobank.org/033D9A5B-9C55-4EBE-B3A3-328F128C815A

##### Holotype male.

**Costa Rica**, Puntarenas province, Monteverde, Estación Biológica Monteverde, 10°19'06.9"N, 084°48'29.3"W, 1530 m, 26.x.2001 (adult emergence), col./rear: Kenji Nishida; host plant: *Hampea appendiculata* (Malvaceae), gall inducer on young leaf veins, pupate outside of the gall, cocoon spun 20.x.2001; RMNH Lepidoptera, Genitalia slide EvN3672, RMNH.INS.23672 (RMNH).

##### Differential diagnosis.

A large rather uniform moth, shining fuscous brown on almost all parts, with an orange frontal tuft and edged eye caps.

##### Description.

**Figures 1–8.**
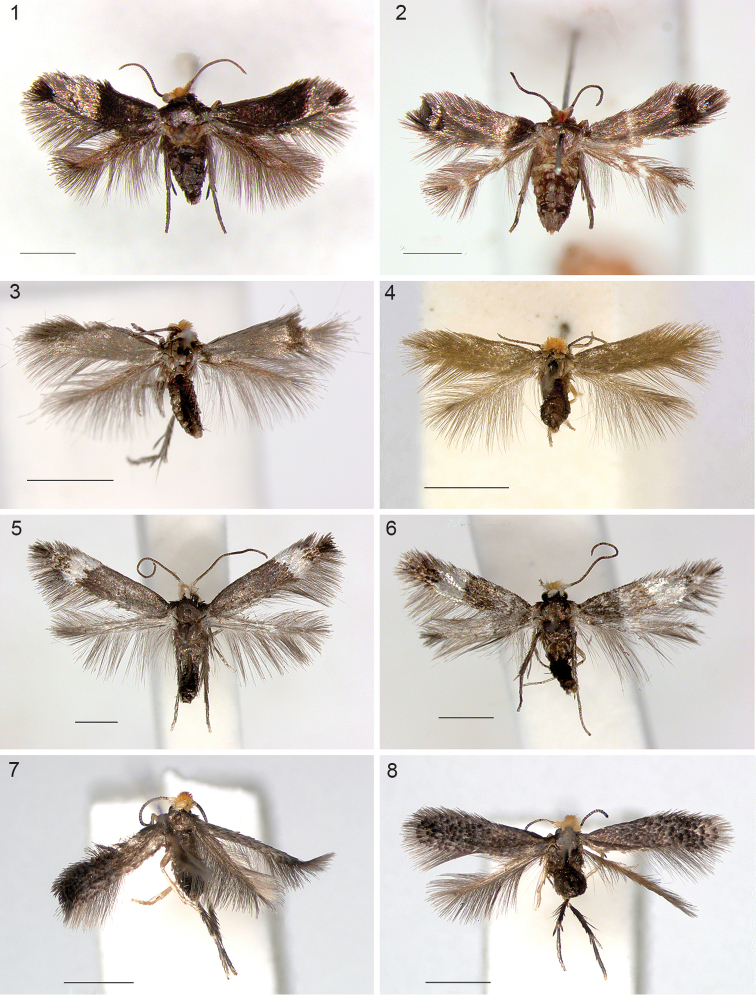
*Stigmella* species, adult habitus. **1** *Stigmella gallicola*, male holotype **2** *Stigmella gallicola*, female Heredia Province, Puerto Viejo de Sarapiquí **3** *Stigmella schinivora*, male holotype **4** *Stigmella schinivora*, female RMNH.INS.24681 **5** *Stigmella costaricensis*, male holotype **6** *Stigmella intronia*, male holotype **7** *Stigmella molinensis*, male holotype **8** *Stigmella molinensis*, female RMNH.INS.24219. Scale bars: 1 mm.

*Male* (Figs 1, 33). Head: frontal tuft orange; antenna with 33–34 segments (n=2); scape cream, with broad grey edge, flagellum brown. Thorax, forewings and hindwings, including fringe, fuscous brown, with strong reflections on forewing and thorax. Abdomen also fuscous, no anal tufts. Legs brown.

*Female* (Fig. 2). Antenna with 30 (n=2), ovipositor slightly protruding, otherwise as male.

*Measurements*. Male: forewing length 2.5 mm (HT), wingspan: 6.0 mm. Female: forewing length 2.5 mm (n=2), wingspan *ca.* 5.8 mm.

**Figures 9–13.**
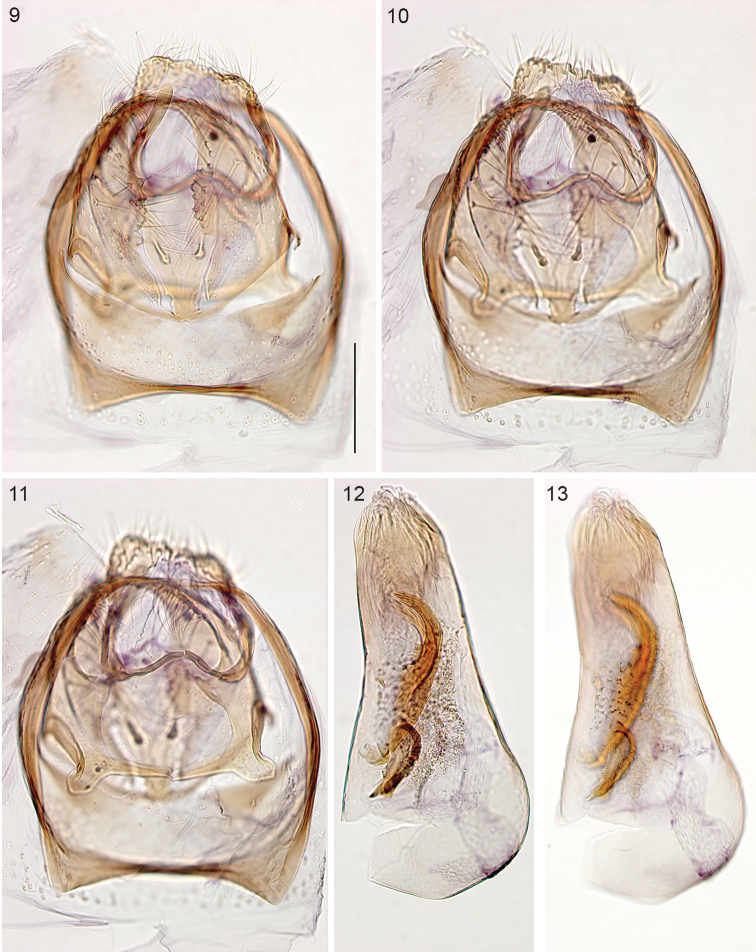
*Stigmella gallicola*, holotype, male genitalia at different levels of focus, phallus separate (**12, 13**). Scale bars: 100 µm, all to same scale.

*Male genitalia* (Figs 9–13). Total length 265–300 µm. Vinculum anteriorly bilobed. Tegumen a narrow band. Uncus rectangular, posterior edge indented to almost straight, with many setae. Gnathos with two curved posterior processes, widely apart, transverse bar curved. Valva length 175–200 µm, more or less triangular, pointed tip slightly curved inward, inner margin serrate in middle, an inward pointed process on dorsal surface; transtilla with long straight transverse bar. Juxta almost triangular with tip anteriorly between valvae, ventrally two prominent setae. Phallus (Figs 12, 13) length 365–375 µm, flask shaped, widest at base, vesica with two large curved cornuti, 145–150 µm long (measured from base to tip in straight line) and several small denticulate cornuti.

**Figures 14–16.**
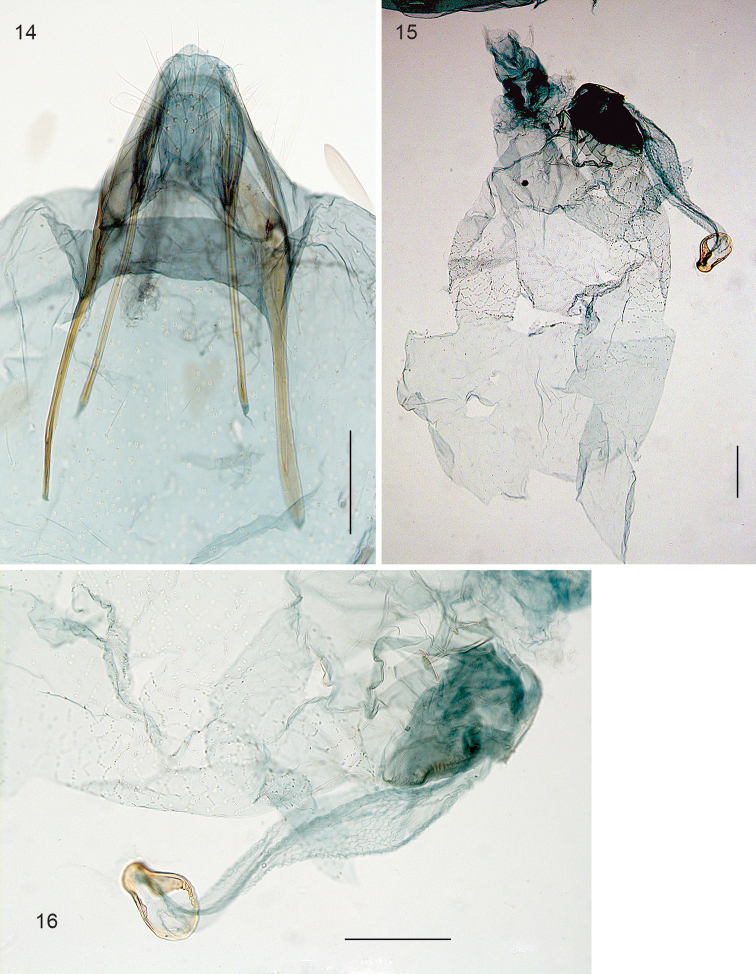
*Stigmella gallicola*, female genitalia, slide EvN3739. **14** Abdominal tip, focussed ventrally **15** Bursa, damaged **16** Detail of 15, with ductus spermathecae and vesicle. Scale bars: 100 µm.

*Female genitalia* (Figs 14–16). Total length of bursa *ca.* 1 mm. Ovipositor pointed (Fig. 14), anterior apophyses longer than posterior ones. T8 narrow, with *ca.* 18 long setae. Bursa covered with pectinations in a reticulate pattern, more prominently in caudal part (Fig. 15). Ductus spermathecae without convolutions, a circular spermathecal vesicle (Fig. 16).

##### Biology.

*Host plant* (Figs 19–20). Malvaceae: *Hampea appendiculata* (Donn.Sm.) Standl. A tree, occurring between Honduras and Panama from sea level to 1800 m on both the Caribbean and Pacific slopes in Costa Rica (Fryxell 2007).

**Figures 17–22.**
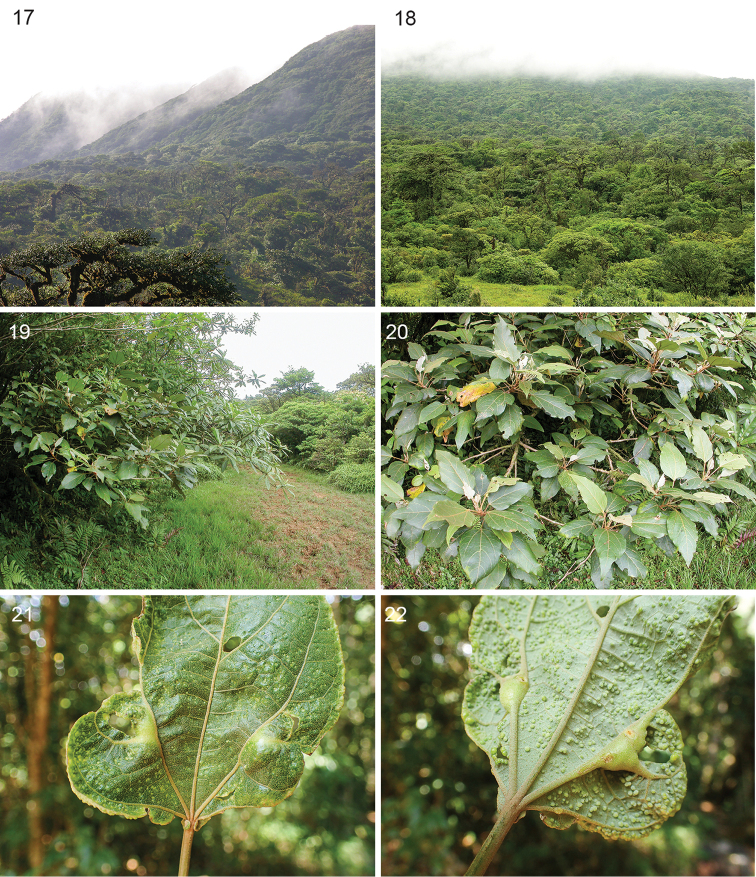
*Stigmella gallicola*, habitat and host plant. **17** General habitat at Alto Masis/Tierras Morenas, a premountain rainforest in Parque Nacional Volcán Tenorio, 13 June 2007 **18** same place, 1 June 2007 **19, 20** Habitat and host, *Hampea appendiculata* at same place, 955 m, 14 June 2007 **21, 22** Mature galls on primary leaf veins, respectively adaxial and abaxial view, Estación Biológica Monteverde 1530 m, 22 April 2016, arrows pointing at nectar glands.

**Figures 23–29.**
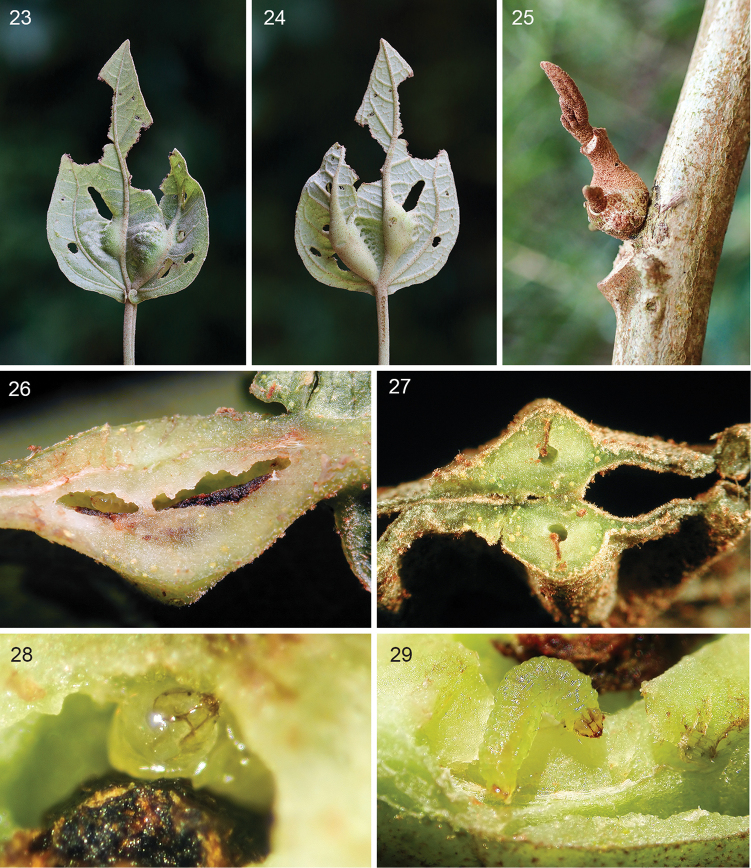
*Stigmella gallicola*, galls and larvae in *Hampea appendiculata*, details, all at Estación Biológica Monteverde, 1530 m. **23, 24** Galls on primary leaf veins of young leaf, respectively adaxial and abaxial view, 8 June 2016 **25** Old gall found at axillary leaf bud, same date **26** Longitudinal cut of mature gall with late instar larva in situ, 20 October 2001 **27** Transversely cut leaf (young gall in middle) showing gall chamber and frass mass line, 24 October 2001 **28** Late instar larva in situ, note packed frass in gall chamber, same date **29** Late instar larva, dorsal view, slightly ex situ after gall dissection, 4 April 2015.

**Figures 30–33.**
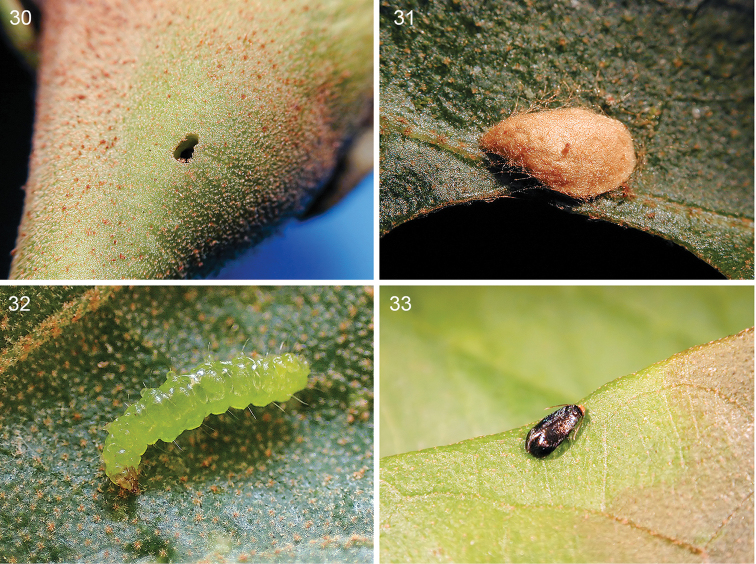
*Stigmella gallicola*, life history details on *Hampea appendiculata*, all at Estación Biológica Monteverde 1530 m. **30** Larval exit hole, 20 October 2001 **31** Cocoon spun on host plant under rearing conditions, same date **32** Recently exited mature larva, 29 April 2016 **33** Reared holotype male resting on host leaf, 26 October 2001.

*Gall* (Figs 21–30). Galls were induced on leaf veins of young leaves and on axillary leaf buds. Galls on leaf veins are ovoid when mature and more swollen on the leaf underside; length 7–18 mm diameter 3–8 mm (n=18). Galls on axillary leaf buds ovoid by swelling of entire buds (Fig. 25) length 8.7– 11.2 mm, diameter 4.1–5.5 mm, gall base of ca. 1.6 mm diameter (n=5). Galls can cause deformation of young leaves. Gall chamber narrow, located longitudinally in central part of gall, and line with compacted dark brown frass on lower part of chamber, part of the frass reaching towards nectary gland (Figs 26, 27). Galls were found on ca. 2 meter-tall treelets or large trees of 8–25 m (n=7), however we were unable to examine higher parts of large trees. Most of the collected galls were empty, having a more or less rectangular-shaped exit slit of less than 1 mm wide (Fig. 30). We found inquiline phorid fly larvae (Diptera: Phoridae) inhabiting old gall chambers. The gall was already recorded by Hanson et al. (2014).

*Egg*. Egg position unknown. Probably laid near or at foliar nectaries (nectar glands) abaxially on primary veins of leaf buds or very young leaves.

*Larva* (Figs 28, 29, 32). Early to late instar translucent (n=7), mature last instar translucent yellowish green, 6.0 mm long (n=1).

*Cocoon* (Fig. 31). Pale brown to brown, oval, exit slit side is broad and opposite side tapered (apple seed-shaped), 2.8–3.2 mm long, 1.7–1.9 mm wide (n=2). Under rearing conditions inside plastic bags, cocoons were constructed on either host leaf surface or on paper towels (n=5). Under natural conditions in the field, no cocoons were found on host plant leaves or stems near the galls.

*Voltinism and habits*. Larvae were found inside the galls in March, May and June. Adult emergence has been recorded in January, April, May, June and October.

*Parasitoids*. Braconidae: Adelinae: *Adelius* sp., endo-parasitoid, koinobiont of host larva and pupating inside host cocoon. It was reared from the Alto Masis Station site (n=2). Adelinae are specialised parasitoids of Nepticulidae (Yu et al. 2011).

##### Distribution.

Costa Rica: Alajuela, Heredia and Puntarenas provinces. Galls have been recorded mostly from Caribbean/Atlantic slope in lowland Costa Rica. The type locality is near the continental divide on the Pacific slope.

##### DNA barcode.

The Holotype (RMNH.INS.23672) and one female (RMNH.INS.23739) are barcoded, with less than 1% difference. Barcode Identification Number BOLD:ACG9386. The female was also sequenced for other genes and used in the molecular phylogeny (Doorenweerd et al. 2016). Sequences may be retrieved in BOLD and Genbank under voucher/sample ID RMNH.INS.23739.

##### Remarks.

The genitalia of *Stigmella gallicola* resemble somewhat those of the *Stigmella betulicola* group, but in our multi-gene molecular analysis it groups in the clade which contains the *Stigmella prunifoliella* and *Stigmella ultima* groups, without a strongly supported position.

There are only few cases of galling in Nepticulidae known, and this species is the first known in the genus *Stigmella*. Galling has evolved independently several times in Nepticulidae: the North American *Ectoedemia populella* Busck, 1907 makes a petiole gall (Busck 1907; Wilkinson and Scoble 1979), but starts with a short leafmine along the midrib. Its behaviour caused Busck to name the new (then monotypic) genus *Ectoedemia* (= making an external swelling), but only a few species feeding on *Populus* have similar habits and all other species in the genus are leafminers. The European *Trifurcula pallidella* (Duponchel, 1843) makes a spindle shaped gall in the stem of *Cytisus* species (Fabaceae) (van Nieukerken et al. 2004), but this gall growth appears to be caused by the intense mining activity in a small portion of the stem. *Muhabettana nigrifasciata* (Walsingham, 1908) of the Canary Islands starts with a small gall on a vein, and later the larva makes a long leafmine in the leaf of *Periploca laevigata* (Apocynaceae) (Klimesch 1972); Klimesch uses the German word “Gallenmine”. *Ectoedemia castaneae* (Busck 1913) was according to Busck reared from small galls on young twigs, but this is probably not a real gall-former, but an effect of a spiral barkmine in a thin branch: the species is synonymised with the barkminer *Zimmermannia bosquella* (Chambers, 1878) (van Nieukerken et al. 2016). Of all these species, *Stigmella gallicola* induces and forms the most conspicuous swellings, and its biology is adapted to live inside the gall chamber. Gall-forming nepticulids could have been overlooked, and further studies in gall-inducing Lepidoptera may reveal more species. It is worth mentioning that larvae of *Rhodoneura* cf. *terminalis* (Walker, 1865) (Thyrididae) induce spindle-shaped galls on the stem apex of *Hampea appendiculata*. They were reared at Estación Biológica Monteverde and OTS La Selva Biological Station (K. Nishida, personal observation).

##### Etymology.

The specific name is a noun in apposition, derived from the Latin noun *galla* (= gall) and suffix –*cola*, “dweller in”.

##### Other material examined.

6♂, 3♀, 5 adults, galls, larvae. **Costa Rica**: 1♂, Heredia Province, Puerto Viejo de Sarapiquí, Trimbina Biological Reserve, 10°24'59.81"N, 084°7'27.82"W, 161 m, gall inducer on young leaf veins of *Hampea appendiculata*, adult emergence18.i.2003, Kenji Nishida, Genitalia slide JCK15024; 2♀, Heredia Province, Puerto Viejo de Sarapiquí, OTS La Selva Station, main bridge, 10°25'53.42"N, 084°0'13.22"W, 50 m, gall inducer on young leaf veins of *Hampea appendiculata*, e.l. 2–5.v.2002, Kenji Nishida, Genitalia slide EvN3739, RMNH.INS.23739; 1♂, Alajuela Province, Parque Nacional Volcán Tenorio, Alto Masis Station, 10°36'39.99"N, 085°0'1.59"W, 955 m, gall maker on *Hampea appendiculata*, young leaf veins, adult emergence 20.vi.2007, Kenji Nishida; 4♂, 1 ♀, Puntarenas Province: Monteverde, Estación Biológica Monteverde, 10°19'06.9"N, 084°48'29.3"W, 1530m, gall inducer on young leaf veins of *Hampea appendiculata*, adult emergence 17.iii–10.iv.2015, cocoons spun approx. 20.iii.2015, Kenji Nishida (RMNH, MZUCR).

##### More data from BOLD

[specimen not examined, same BIN]. 1 adult, **Costa Rica**, Guanacaste, Area de Conservacion Guanacaste, Sector San Cristobal, Estacion San Gerardo, 10.88 -85.389, 575 m, 17–24.ii.2014, DH Janzen, W Hallwachs, Malaise trap GMP#05845, laguna, BIOUG24701-G07 (BIOUG).

### *Stigmella prunifoliella* group

#### Stigmella schinivora

Taxon classificationAnimaliaLepidopteraNepticulidae

van Nieukerken sp. n.

http://zoobank.org/00C3D062-71A9-400B-8A26-E416451048F9

##### Holotype male.

**Argentina** (Misiones), Cataratas del Iguazú. 27.viii.2000, 21J YM5645, E.J. van Nieukerken; leafmines, rather cultivated part of park; *Schinus terebinthifolius*, e.l. 11–12.ix.2000, RMNH/EvN no 2000148–1, Genitalia slide EvN3986, RMNH.INS.23986 (RMNH).

##### Differential diagnosis.

A completely leaden coloured species, with orange head and leaden edged scape, collar also leaden. The genitalia do not resemble any other Neotropical *Stigmella* species closely.

##### Description.

*Male* (Fig. 3). Head with frontal tuft yellow, collar leaden grey, scape white, edged grey posteriorly, pedicel white, flagellum leaden; antenna with 23–24 segments (n=3). Thorax and forewings uniformly shining leaden grey, smoothly scaled, hindwings slightly darker. Underside forewing dark grey. Abdomen without anal tufts.

*Female* (Fig. 4). Antenna with 20–21 segments (n=2). Scape hardly with grey edging, hindwings paler than in male, same colour as forewings.

*Measurements*. Male: forewing length 1.7–1.8 mm (n=2), wingspan: 4.0 mm (n=2). Female: forewing length 1.7– 2.0 mm (n=2), wingspan 3.9–4.2 mm (n=2).

**Figures 34–37.**
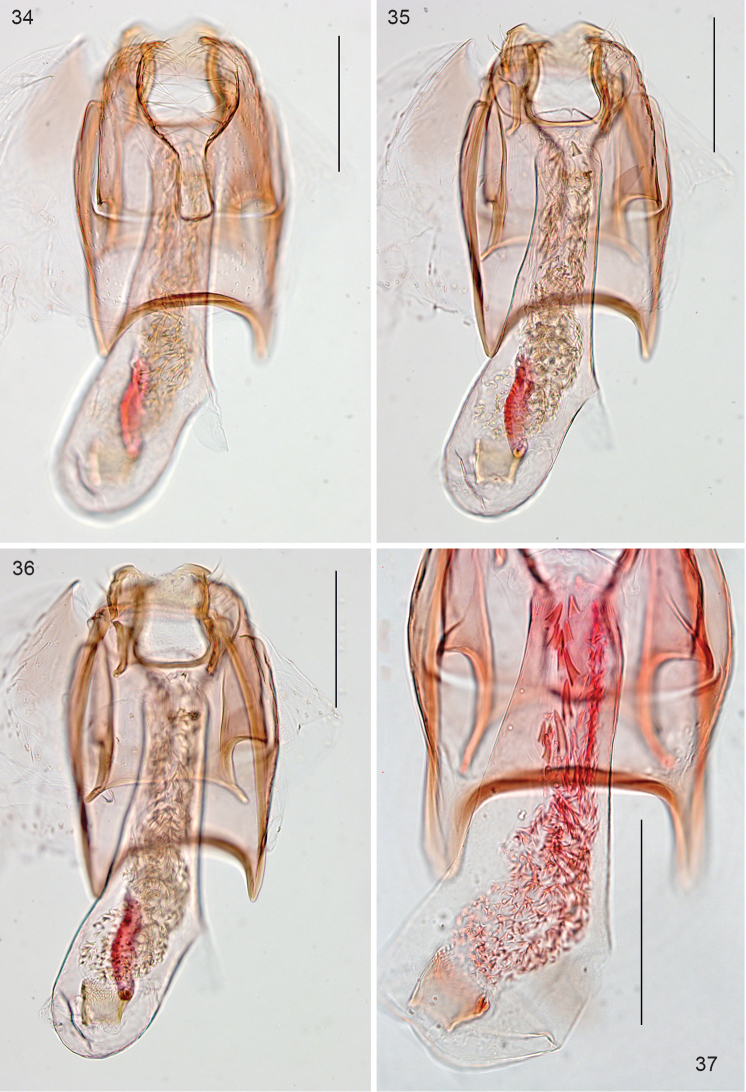
*Stigmella schinivora*, male genitalia. **34–36** Holotype, slide EvN3986, in different levels of focus **37** Detail of cornuti in phallus, slide EvN4500. Scale bars: 100 µm.

*Male genitalia* (Figs 34–37). Total length vinculum 185–200 µm, valva 135µm, phallus 260–285 µm (n=3). Phallus asymmetric, anteriorly bending to the left, vesica with many small cornuti.

**Figures 38–40.**
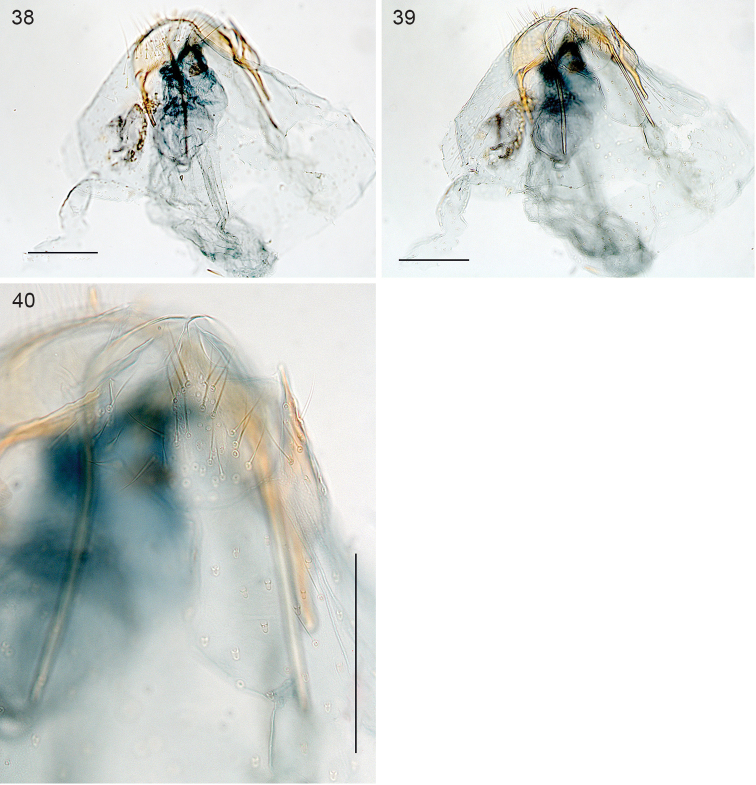
*Stigmella schinivora*, female genitalia, slide EvN4681 **38, 39** Overview at different focus levels **40** Details of tergum 8. Scale bars: 100 µm.

*Female genitalia* (Figs 38–40). Length of bursa ca 340 µm. T8 apically pointed, with ca 15–20 setae total. Posterior apophyses longer than anterior ones. No sclerotisations or signa observed in the single relatively poor genitalia slide.

##### Biology.

*Host plants*. Anacardiaceae: *Schinus terebinthifolia* Raddi, the Brazilian pepper tree.

**Figures 41–43.**
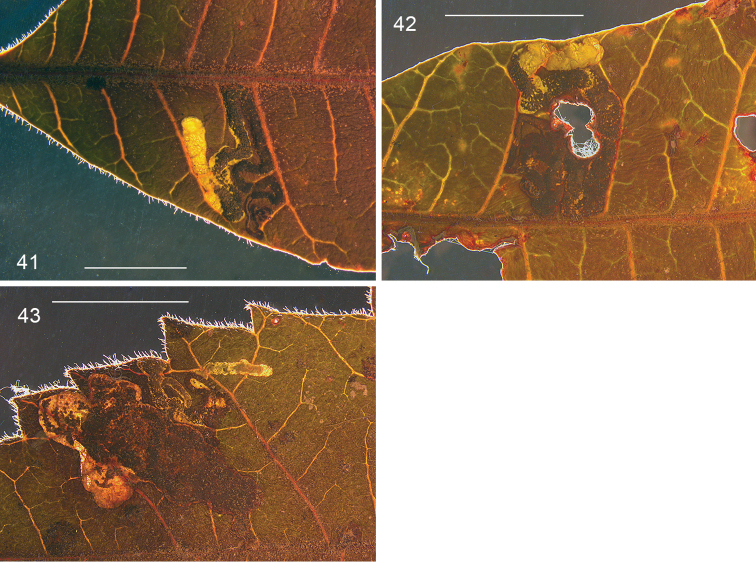
*Stigmella schinivora*, leafmines on *Schinus terebinthifolia*. **41** EvN 2000148H, collected as vacated mine **42** EvN 2000148K, mine from which larva was reared **43** EvN 2000149K, mine with dead larva, next to a mine of *Leurocephala schinusae*, the large brown blotch. Scale bars: 5 mm.

*Leafmines* (Figs 41–43). The mine is a much contorted upper surface gallery, track often doubling back, usually confined to the small space between two lateral veins and the midrib; mine filled with black frass; larval exit hole on upperside. Mine poorly visible in transmitted light, or from underside, due to leaf thickness.

*Egg*. The egg is always deposited on leaf upperside, frequently against a lateral vein.

*Larva*: green.

*Voltinism and habits*. Larvae were collected in late August, adults emerged indoors in September, and were caught in malaise traps from late September to late October. We collected the mines on planted trees, together with large numbers of the then still undescribed *Leurocephala schinusae* Davis & McKay (Davis et al. 2011).

##### Distribution.

Argentina: Misiones.

##### DNA barcode.

We barcoded two specimens from our reared series (not the Holotype), that appeared to match with a large number of records from the Malaise traps in Misiones, giving a total of 26 barcodes, all in BIN BOLD:ACN0764. One specimen was also sequenced for other genes and will be used in a forthcoming analytical paper. Sequences may be retrieved in BOLD and later also in Genbank under voucher/sample ID RMNH.INS.24681.

##### Remarks.

Molecular analysis suggest that *Stigmella schinivora* is closely related to the North American Anacardiaceae feeding species in the *Stigmella prunifoliella* group, which suggests a single host-shift from Rosaceae or Rhamnaceae, but in some analyses it also groups with the European *Stigmella diniensis* (Klimesch, 1975) that feeds on Cistaceae. It is currently the only known Neotropical species of this group of species.

Davis et al. (2011) described four gracillariid leafminers of the Brazilian pepper tree, partly aiming at finding suitable candidates to release as biological control against the invading pepper tree in Florida. They did not report on any nepticulid, but we assume that *Stigmella schinivora* is also widespread with the host. It could be added to the list of potential control candidates, but we doubt its effectiveness, given the small size. Also the fact that several Anacardiaceae miners are not very host specific is a risk, as released control species might shift to native North American *Rhus* and *Toxicodendron* species.

##### Etymology.

Schinivora: an adjective, derived from the Latin noun *Schinus* (host genus), stem schin-, and verb *voro* (=devour).

##### Other material examined.

4♂, 3♀, 4 larvae, leafmines. **Argentina**: 1♂, 1♀, 3 larvae in alcohol, leafmines, Misiones, Cataratas del Iguazú. 27.viii. 2000, leafmines, rather cultivated part of park; leafmines on *Schinus terebinthifolia*, e.l. 11–12.ix.2000, RMNH/EvN no 2000148–1, E.J. van Nieukerken; Genitalia slides EvN3986, RMNH.INS.23986 (RMNH); 1♂, 1♀, 1 larva in alcohol, leafmines, same locality, but e.l. 14–15.ix.2000, RMNH/EvN no 2000149–1, Genitalia slides ♂ EvN4500, ♀ EvN4681, RMNH.INS.24500, 24681 (RMNH); 1♂, 1♀ , in ethanol 96%, Misiones, Obera, CIAR, 26.ix.–3.x.2013, Pablo Tubaro, Malaise trap, GMP#05155, -27.445, -54.94, 147 m, DNA-barcoded, BIOUG13587-H11 & BIOUG13589-E08 (MACN), 1♂, ditto, but 24–31.x.2013, GMP#05157, BIOUG13956-F02, whole specimen mounted on slide, EvN4834 (MACN).

##### More data from BOLD

[specimens not examined, same BIN]. 21 adults, in ethanol 96%, Argentina, Misiones, Obera, CIAR, 23.v.–24.x.2013, Pablo Tubaro, Malaise trap: 1, trap GMP#05146, 23–30.v.2013, BIOUG12989-H09; 2 ad., trap GMP#05149, 26–4.vii.2013, BIOUG23823-E01, BIOUG23823-E11; 2 ad., trap GMP#05150, 11–18.vii.2013, BIOUG24501-C12, BIOUG24505-B08; 1 ad., trap GMP#04812, 18–25.vii.2013, BIOUG13317-B04; 4 ad., trap GMP#05151, 25–1.viii.2013, BIOUG24746-D11, BIOUG24746-E04, BIOUG24746-E06, BIOUG24746-H10; 5 ad., trap GMP#04813, 1–8.viii.2013, BIOUG13418-F06, BIOUG13418-G07, BIOUG13418-H11, BIOUG13420-A11, BIOUG13421-G02; 1 ad., trap GMP#04815, 22–29.viii.2013, BIOUG24913-G01; 2 ad., trap GMP#04816, 5–12.ix.2013, BIOUG25104-F01, BIOUG25104-F02; 1 ad., trap GMP#04817, 19–26.ix.2013, BIOUG25154-H01; 2 ad., trap GMP#04819, 17–24.x.2013, BIOUG24948-E10, BIOUG24952-A07 (all in MACN).

#### Stigmella costaricensis

Taxon classificationAnimaliaLepidopteraNepticulidae

van Nieukerken & Nishida sp. n.

http://zoobank.org/DA4DC25D-B3CC-4ACE-9B46-18992520103F

##### Holotype male.

**Costa Rica**, San José Province, Parque Nacional Chirripó, Llano Bonito, Refugio, 09°27'16"N, 083°32'41"W, 2492 m, 19–20.ii.2009, Light, Leg: Kenji Nishida. Genitalia slide EvN4037♂, RMNH.INS.24037 (RMNH).

##### Differential diagnosis.

See under *Stigmella intronia*.

##### Description.

*Male* (Fig. 5). Head: frontal tuft yellow, collar white, scale and pedicel white, flagellum grey brown. Antenna with 45 segments. Thorax, legs, forewing and hindwing grey brown, with slight iridescence, cilia similarly coloured; a shining white fascia at 2/3, width ca 1/5 of wing length, wider at costa than at dorsum. Abdomen as thorax, no anal tufts.

*Female*. Unknown.

*Measurements*. Male: forewing length 3.5 mm (n=1), wingspan: ca 7.5 mm.

**Figures 44–47.**
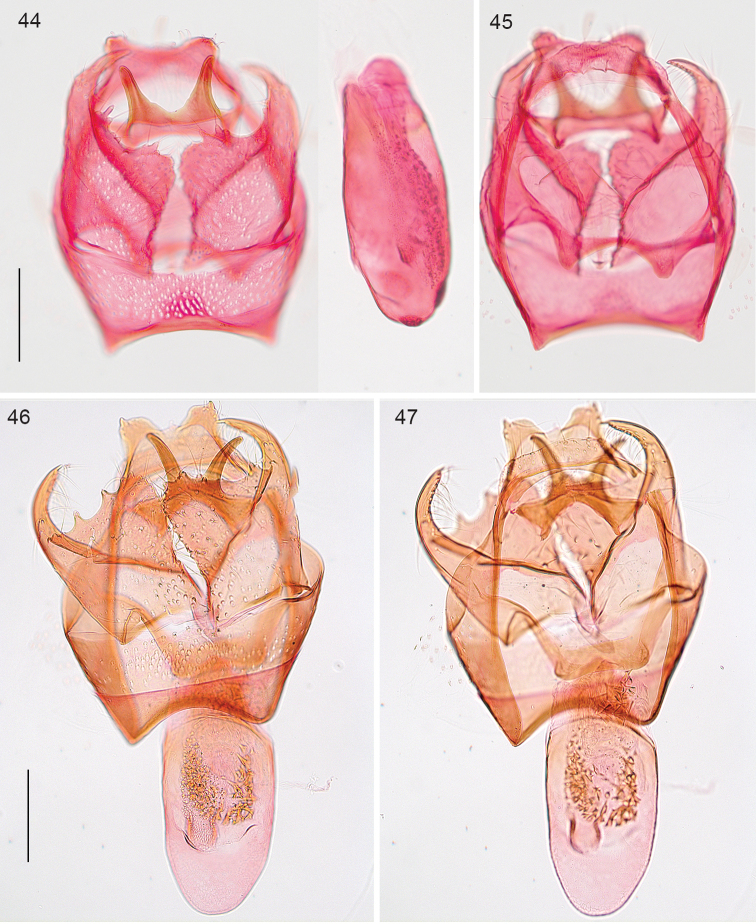
*Stigmella* species, male genitalia. **44, 45** *Stigmella costaricensis*, holotype **46, 47** *Stigmella intronia*, holotype. Scale bars: 100 µm.

*Male genitalia* (Figs 44, 45). Total length capsule 305µm. Uncus deeply bilobed. Gnathos with posterior horns closely placed, diverging posteriorly. Valva length ca 225 µm, almost squarish with prominent curved distal process, posterior edge deeply serrate by setal sockets, internal edge almost straight, with slight notch; transtilla without sublateral processes. Phallus length ca 260 µm, tubular, no carinae or juxta present; vesica with many small cornuti.

##### Biology.

*Host plants*. unknown.

*Voltinism and habits*. The moth was collected in February at a light sheet.

##### Distribution.

Costa Rica: San José Province: Chirripó National Park: Llano Bonito area, a cloud forest surrounded by large oak trees.

##### DNA barcode.

Holotype BIN: BOLD:ACG8765. The holotype was also sequenced for three other genes and will be used in a forthcoming analytical paper. Sequences may be retrieved in BOLD and later also in Genbank under voucher/sample ID RMNH.INS.24037.

##### Remarks.

This species and the next were collected the same night. They both belong in core *Stigmella*, in the clade with the *Stigmella lapponica*, *Stigmella rhamnella* and *Stigmella sanguisorbae* groups, but do not belong to any of these groups.

##### Etymology.

Costaricensis: an adjective, derived from the country name Costa Rica and the suffix –*ensis*, indicating geographical origin.

#### Stigmella intronia

Taxon classificationAnimaliaLepidopteraNepticulidae

van Nieukerken & Nishida sp. n.

http://zoobank.org/45DAED60-C96C-48D5-B074-EE768B49380E

##### Holotype male.

**Costa Rica**, San José Province, Parque Nacional Chirripó, Llano Bonito, Refugio, 09°27'16"N– 083°32'41"W, 2492 m, 19–20.ii.2009, Light, Leg: Kenji Nishida. Genitalia slide EvN4036♂, RMNH.INS.24036 (RMNH).

##### Differential diagnosis.

Externally *Stigmella intronia* and *Stigmella costaricensis* are very similar, but the fascia in *intronia* seems a bit wider and is placed more anteriorly. Both species resemble somewhat the North American *Stigmella slingerlandella* (Kearfott, 1908). The species are best separated by the shape and spinosity of the valva and form of uncus and gnathos.

##### Description.

*Male* (Fig. 6). Head: frontal tuft yellow, collar white, scape and pedicel white, flagellum grey brown. Antenna with 44 segments. Thorax, legs, forewing and hindwing grey brown, with slight iridescence, cilia similarly coloured; a shining white fascia at 1/2, width ca. 1/4 of wing length, wider at dorsum than at costa. Abdomen as thorax, no anal tufts.

*Female*. Unknown.

*Measurements*. Male: forewing length 2.8 mm (n=1), wingspan: ca 6.3 mm.

*Male genitalia* (Figs 46, 47). Total length capsule 300 µm. Uncus bilobed, lobes far apart. Gnathos with posterior horns separate, almost parallel. Valva length ca 240 µm, somewhat squarish, with prominent curved distal process, posterior edge partly serrate by setal sockets, internal edge curved outwards; transtilla with sublateral processes extremely short to almost absent. Phallus length ca 290 µm, tubular, no carinae or juxta present; vesica with many small cornuti.

##### Biology.

*Host plants*. Unknown.

*Voltinism and habits*. The moth was collected in February at a light sheet.

##### Distribution.

Costa Rica: San José Province: Chirripó National Park: Llano Bonito area, a cloud forest surrounded by large oak trees.

##### DNA barcode.

Holotype BIN: BOLD:ACG8514. The holotype was also sequenced for other genes and used in the molecular phylogeny (Doorenweerd et al. 2016); here we discovered the presence of several introns in a copy of the gene Elongation Factor 1α. Sequences may be retrieved in BOLD and Genbank under voucher/sample ID RMNH.INS.24036.

##### Remarks.

See under *Stigmella costaricensis*.

##### Etymology.

Intronia, a noun in apposition, arbitrarily derived from the word Intron (based on English: intragenic region), because of the presence of several introns in a copy of the gene Elongation Factor 1α (Doorenweerd et al. 2016).

### *Stigmella salicis* group

#### Stigmella molinensis

Taxon classificationAnimaliaLepidopteraNepticulidae

van Nieukerken & Snyers sp. n.

http://zoobank.org/BD31E11A-6C3A-4D7D-9F9B-88677E9735BB

##### Holotype male.

**Peru**, Lima, Universidad Agraria la Molina, 240 m, -12.0869, -76.9444, 1.xii.2010, leafmines on *Salix humboldtiana*, e.l. 16.xii.2010, C. Snyers, genitalia slide EvN4218, RMNH.INS.24218 (UNALM).

##### Differential diagnosis.

Externally *Stigmella molinensis* can be confused with other Neotropical *Stigmella* without fascia, with pale head and collar, such as *Stigmella hamata* Puplesis & Robinson, 2000, *Stigmella montanotropica* Puplesis & Diškus, 2002 and *Stigmella austroamericana* Puplesis & Diškus, 2002, but the male genitalia are characteristic.

##### Description.

*Male* (Figs 7, 64, 65). Head: frontal tuft orange, collar and scape yellowish white, flagellum brown. Antenna with 24 segments. Thorax and forewing fuscous, with dark tipped scales, a more or less distinct cilia line, demarcating the grey fringe; forewing without pale spots. Hindwing narrow, grey. Hindlegs fuscous with white parts, other legs yellowish white.

*Female* (Fig. 8). Antenna with 19–23 segments (n=4).

*Measurements*. Male: forewing length ca 1.9–2.0 mm (n=2), wingspan: 4.4–4.5 mm. Female: forewing length 2.3 mm, wingspan 5.1 mm.

**Figures 48–53.**
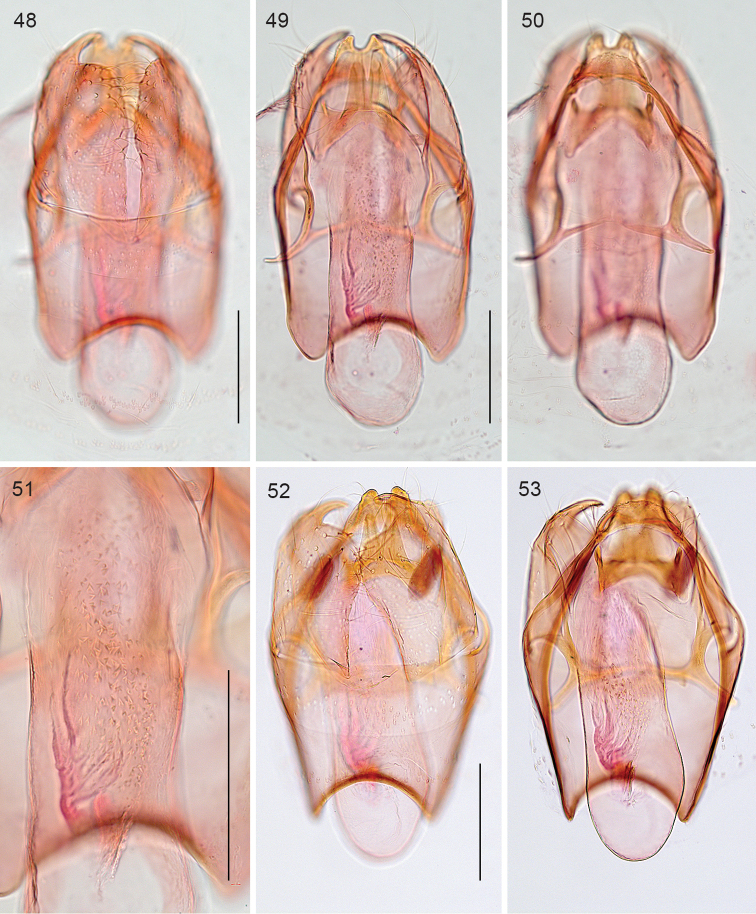
*Stigmella molinensis*, male genitalia. **48–51** slide EvN4812 **52–53** Holotype, slide EvN4218. Scale bars:100 µm.

*Male genitalia* (Figs 48–53). Total length capsule 240 µm (n=2). Uncus narrow, bilobed, with deep medial notch. Gnathos narrow, with posterior processes fused basally, distally just separate. Valva 155–170 µm long, with short pointed and curved distal process, inner lobe broad, rounded, with almost straight, parallel inner margins; transtilla with sublateral processes ca half as long as transverse bar. Phallus length 250–260 µm, vesica with many small denticulate cornuti.

**Figures 54–57.**
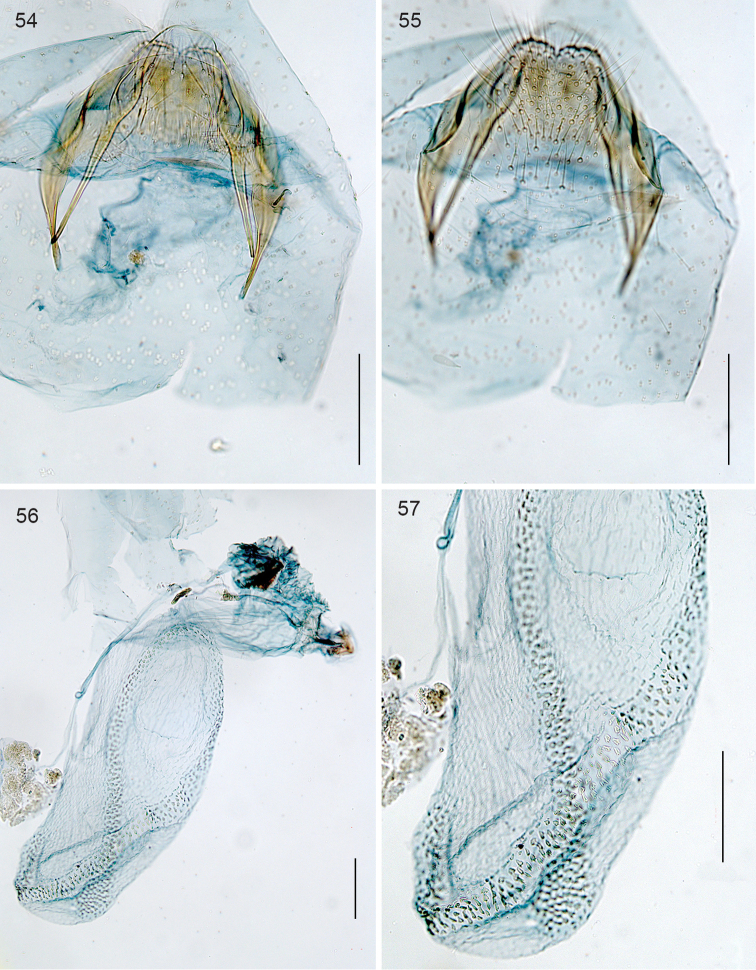
*Stigmella molinensis*, female genitalia, slide EvN4219. **54, 55** Terminal segments, respectively focussed dorsally, showing T8 and ventrally, showing setae on S8 **56, 57** Bursa and detail. Scale bars:100 µm.

*Female genitalia* (Figs 54–57). Total length bursa ca 730 µm. Abdominal tip rather narrow, but not pointed; T8 with 5–7 setae on either side, and a small patch of many small setae anteriorly; anal papillae without setae. Bursa covered with small pectinations, with a band of larger and stronger sclerotised ones, in 4 rows, running around bursa longitudinally; ductus spermathecae straight, without convolutions.

##### Biology.

**Figures 58–65.**
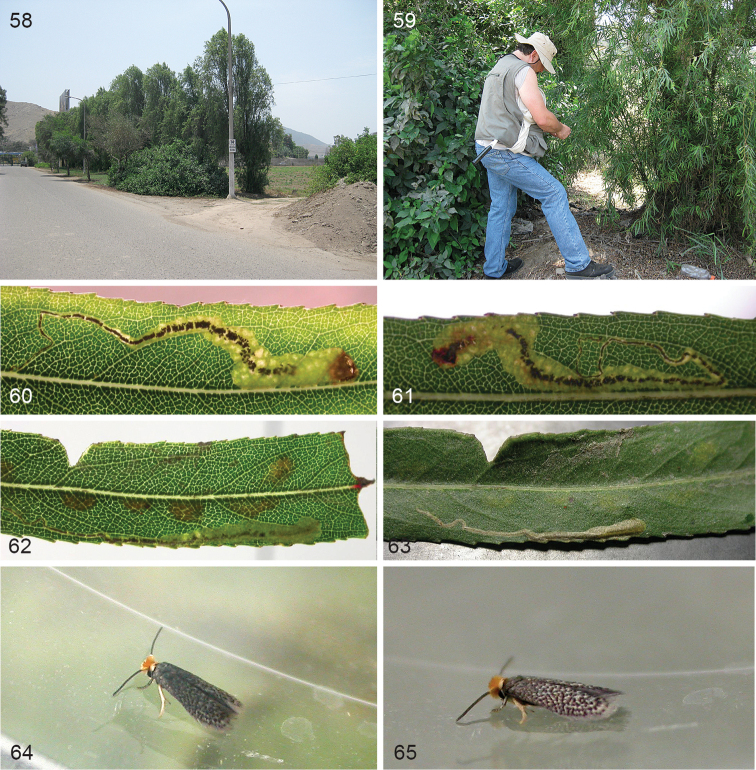
*Stigmella molinensis*, type locality Lima, Universidad Agraria la Molina, 240 m, host plant and life history. **58** Row of tall *Salix humboldtiana* trees, 9 December 2013 **59** Chris Snyers, collecting leafmines in *Salix humboldtiana*, 16 December 2013 **60–63** Vacated leafmines and leafmine with larva, 18 January 2010 **64–65** Male, emerged 18 January 2010.

*Host plant* (Figs 58, 59). Salicaceae: *Salix humboldtiana* Willd., a small tree.

*Leafmines* (Figs 60–63). Mine first a narrow gallery, usually running towards leaf edge and leaf apex and not doubling back, occasionally running to leaf base and then often doubling back and ending towards apex. Later mine a wide irregular gallery, almost full depth. Frass black, in a narrow central line. larval exit hole on leaf upperside.

*Egg*. Always deposited on leaf upperside close to the midrib.

*Larva* yellow.

*Voltinism and habits*. Larvae were always present between December and late January. Adults usually emerged after two to three weeks after the cocoon was spun, suggesting multiple generations. The species has only been searched for between early December and late January, we have no information on other seasons. Mines were always found at the shady side of the trees.

##### Distribution.

Peru: Lima.

##### DNA barcode.

We barcoded three specimens: the Holotype, the female RMNH.INS.24219 and the male RMNH.INS.24812. BIN: BOLD:ACG9223. The female was also sequenced for other genes and used in the molecular phylogeny (Doorenweerd et al. 2016). Sequences may be retrieved in BOLD and Genbank under voucher/sample ID RMNH.INS.24812.

##### Remarks.

This species clearly belongs to the *Stigmella salicis* group s.str., that is widespread in the Holarctic region and of which all but one species feed on Salicaceae (sensu stricto). However, morphologically it differs by the presence of numerous small cornuti in the phallus, whereas all other species have a reduced number of differently shaped cornuti; the latter thus is a good apomorphy for the Holarctic members of the group. The female shares the characteristic apomorphy: a band of signa around the bursa copulatrix. In our molecular phylogeny (Doorenweerd et al. 2016) *Stigmella molinensis* is sister to all Holarctic species of the group. The other Neotropical species, included in the *salicis* group by Puplesis and Robinson (2000) are excluded by us and placed in the *Stigmella epicosma* group of which probably many feed on Asteraceae. Since the host plant, *Salix humboldtiana*, and ten other native *Salix* species (Alford and Belyaeva 2009) are widespread in South and Central America, we expect that *Stigmella molinensis* and possibly related species are more widespread.

##### Etymology.

The specific name, an adjective, is derived from La Molina, Spanish and Latin for Mill, and also the name of the district in Lima and the University where the species was collected.

##### Other material examined.

Adults and leafmines: Same data as Holotype, **Peru**, 1♂, 18.i.2010, e.l. 1.ii.2010, damaged specimen (see Figs 64, 65); 1♀, Genitalia slide EvN4219, RMNH.INS.24219; 3♀, xii.2013, e.l. xii.2013; 1♀ i.2013, e.l. i.2013; 1♂, 1♀, 15.xii.2014, e.l. i. 2015, ♂ Genitalia slide EvN4812, RMNH.INS.24812 (all RMNH).

#### Ozadelpha

Taxon classificationAnimaliaLepidopteraNepticulidae

van Nieukerken gen. n.

http://zoobank.org/C69504F8-9528-480A-806A-BEAE4B2E0DB8

##### Type species.

*Ozadelpha conostegiae* van Nieukerken & Nishida, sp. n. by present designation.

##### Differential diagnosis.

*Ozadelpha* is recognised by the collar with lamellar scales (as in *Enteucha* and *Stigmella*), usually two fascia’s on forewing; forewing venation without closed cell, but usually a very long separate CuA. Male genitalia are characterised by separate vinculum and tegumen, large vinculum, bilobed uncus and V-shaped gnathos.

##### Description.

**Figures 66–67.**
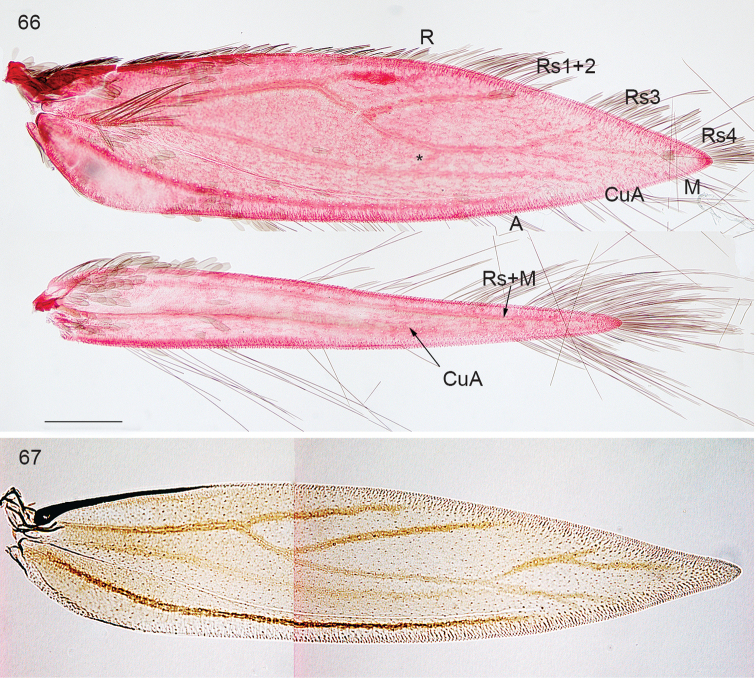
*Ozadelpha* gen. n., venation. **66** *Ozadelpha conostegiae*, female, slide EvN4704, veins labelled **67** *Ozadelpha ovata*, paratype male, forewing only, slide AD207. * this is probably a staining artefact, not a cross vein, Scale: 200 µm.

**Figures 68–69.**
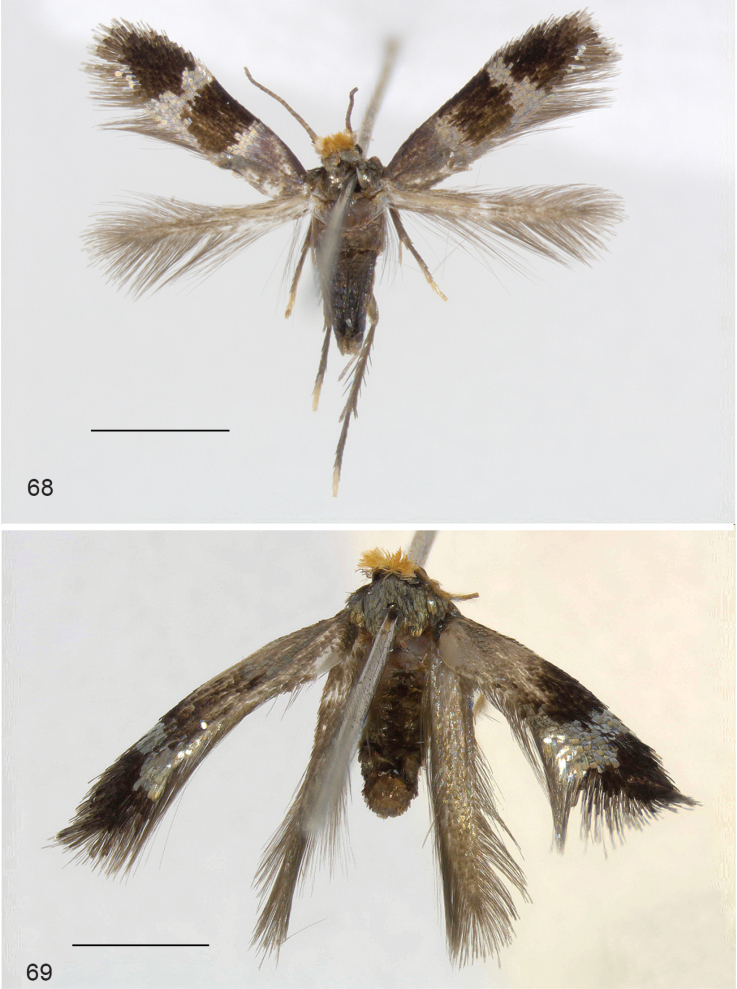
*Ozadelpha* gen. n., adult habitus. **68** *Ozadelpha conostegiae*, holotype male **69** *Ozadelpha* specimen EvN4680, female. Scale bars:1 mm.

Adult (Figs 68, 69). Rather small nepticulid moths, forewing length 1.4–2.8 mm. Head with collar comprising lamellar scales, but piliform in *Ozadelpha ovata*. Antenna with 21–32 segments in male, 19 in female (no more data available). Forewing usually with two pale, often metallic fasciae, sometimes joined along dorsal margin. Hindwing in male sometimes with androconial scales on wing (in *Ozadelpha guajavae* and *Ozadelpha ovata*) or a hairpencil near frenulum (*Ozadelpha conostegiae*). Venation (Figs 66, 67): R+Rs+M coalescent from base, kinked at junction of R, with 5 branches: R, Rs1+2, Rs3, Rs4 and M, CuA separate and long, approaching Rs+M (what looks like a cross-vein between CuA and Rs+M in Fig. 68 is probably an artefact), absent in the drawing of *Ozadelpha guajavae* (Puplesis et al. 2002a); A thickened; Hindwing very narrow, with 2 veins only (Rs+M and CuA), Rs+M close to costa.

**Figures 70–73.**
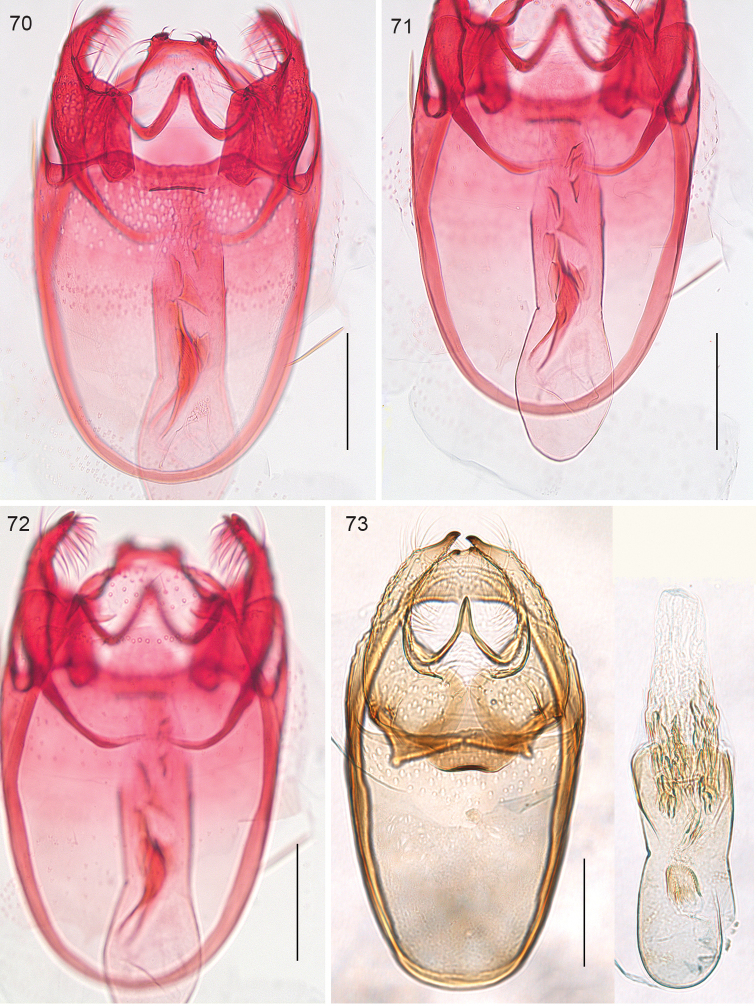
*Ozadelpha* gen. n., male genitalia. **70–72** *Ozadelpha conostegiae*, holotype, at different levels of focus **73** *Ozadelpha ovata*, holotype. Scale bars: 100 µm.

*Male genitalia*. (Figs 70–73). Vinculum lateral arms articulate with sides of tegumen; ventral plate expanded, not bilobed. Tegumen band shaped. Uncus variously bilobed. Gnathos an inverted V. Valva more or less triangular, transtilla with transverse bar present or weakly sclerotised, sublateral processes absent or short. Phallus relatively short, without distinct carinal processes; cathrema a normal striate thickening around base of ejaculatory duct, vesica with variable number of cornuti.

**Figures 74–79.**
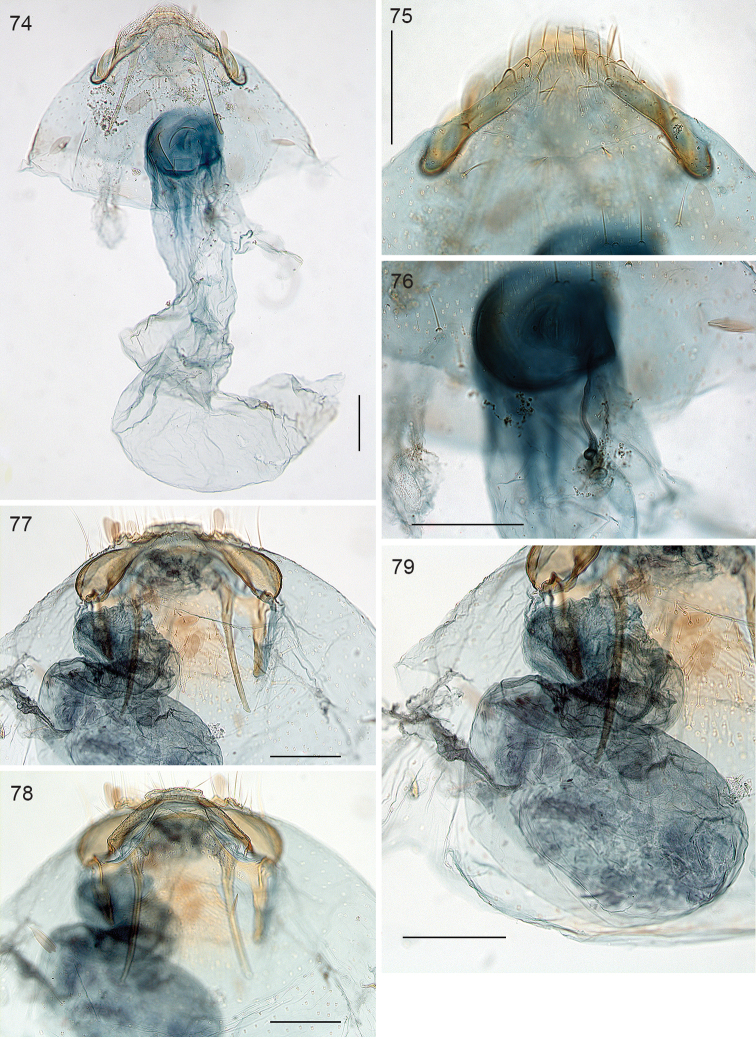
*Ozadelpha* gen. n., female genitalia. **74** *Ozadelpha conostegiae*, dorsal view, slide 4847 **75** ditto, detail of T8 in dorsal view **76** ditto, detail of ductus spermathecae **77–79** *Ozadelpha* specimen EvN4680. Scale bars: 100 µm.

*Female genitalia* (Figs 74–79). T8 with or without row of setae; no setose anal papillae. Anterior apophyses often broadened, posterior apophyses usually narrow, straight, and longer than anterior ones. Vestibulum folded, more strongly staining in Chlorazol Black, with indistinct or no sclerotizations; corpus bursae asymmetric, curved; wall completely devoid of spines or pectinations or with a group of numerous, large, blunt pectinations as in *Ozadelpha guajavae* (Remeikis et al. 2014). Ductus spermathecae with 1–3.5 convolutions.

*Larva*. Green, where known.

##### Biology.

Leafminers on Melastomataceae and Myrtaceae, both belonging to the order Myrtales. See also Remeikis et al. (2014).

##### Distribution.

Central and South America, known from Costa Rica, Ecuador and Argentina, probably widespread.

##### Composition.

We describe here one new species, *Ozadelpha conostegiae*, as type species, plus a second one from the same host that we do not name. Both *Stigmella ovata* Puplesis & Robinson, 2000 and *Enteucha guajavae* Puplesis & Diškus, 2002 share many characters with the type species: an almost identical venation (albeit without recognisable CuA in the drawing of *Enteucha guajavae*) and very similar male genitalia. We therefore transfer both to this genus as *Ozadelpha ovata* (Puplesis & Robinson, 2000), comb. n. and *Ozadelpha guajavae* (Puplesis & Diškus, 2002), comb. n. It is possible that some species associated with the Myrtaceae genus *Myrceugenia* from Patagonia (Nielsen 1985) belong here as well, although we observe some differences in the few specimens available to us. The Vilnius group is planning to revise this group of species (J.R. Stonis, personal communication).

##### Etymology.

*Ozadelpha*, a noun. The name is based on a combination of the Greek adelphe (αδελφη), meaning sister with the colloquial abbreviation “Oz” as often used for Australia. This to indicate the sister group relationship between *Ozadelpha* and the Australian clade with *Roscidotoga* Hoare, 2000, *Pectinivalva* Scoble, 1983, *Menurella* Hoare, 2013 and *Casanovula* Hoare, 2013. The name is to be treated as feminine.

##### Remarks.

The series reared from *Conostegia* that KN reared and sent via the Museo de Zoología, Universidad de Costa Rica, to EvN was at first considered to constitute a single species. We were only able to successfully amplify several genes from one large female (RMNH.INS.24680) that on closer inspection appeared to be different from the rest of the series, even though the differences externally are small, apart from the size. The position of the genus as sister of the Australian genera was strongly supported on the basis of this specimen; however, we do not name that by lack of a male specimen and life history data. The few genes that we did amplify from other specimens show that both species belong to the same clade, and thus the same genus. In fact, there are probably more species feeding on different species of *Conostegia* throughout its distribution area; just before finalising this manuscript, Kenji Nishida reared another species from another species of *Conostegia*.

In the morphology *Ozadelpha* shows similarities with both *Stigmella*, *Enteucha* and the Australian genera. The venation resembles *Stigmella*, but also *Pectinivalva*, apart for the thickened A in forewing. In our molecular phylogeny *Ozadelpha* always groups with the Australian genera, either as sister to all of them together, or as sister to *Roscidotoga* (Doorenweerd et al. 2016). Where both *Ozadelpha* and *Pectinivalva* in the old sense feed on Myrtales, it is possible that the ancestral hosts were also Myrtales and that the ancestor was a rainforest inhabitant (Hoare and van Nieukerken 2013).

#### Ozadelpha conostegiae

Taxon classificationAnimaliaLepidopteraNepticulidae

van Nieukerken & Nishida sp. n.

http://zoobank.org/40CDDC2D-0672-4289-B525-03CCBBFC8FEC

##### Holotype male.

**Costa Rica**, Puntarenas Province, Monteverde, Estación Biológica Monteverde, 10°19'06.9"N, 084°48'29.3"W, 1530 m, 2.iii.2012 collected leafmines, 31.iii.2012 adult emergence, host plant: *Conostegia oerstediana* (Melastomataceae), photos/leg/rear: Kenji Nishida, RMNH Lepidoptera, Genitalia slide EvN4506, RMNH.INS.24506 (RMNH).

##### Differential diagnosis.

Externally recognised by leaden collar of lamellar scales and forewing with two fasciae, the first indistinctly joined to basal leaden area. Male genitalia unique and can not be confused with other Nepticulidae. However, there are as yet unnamed rather similar species. See also next species.

##### Description.

*Male* (Figs 68, 89). Head: frontal tuft yellow orange, scape white; antenna with 24–25 segments (n=2). Collar comprising lamellar scales, leaden. Thorax and forewings dark fuscous, forewing with basal leaden patch, poorly separated from silver fascia at 1/3, a second silver fascia at 2/3, often broken or narrowed in middle, at dorsum widened in both directions, silver scales along dorsum may reach other fascia, usually not; more distal silver scales usually separated from fascia. Hindwing with a narrow ochreous hairpencil inserted near frenulum of 1/3 hindwing length, hindwing scaling brown-grey. Leg upperside and abdomen dark fuscous, tarsi paler. Abdomen with indistinct grey anal tufts.

*Female* (Fig. 90). Antenna with 19 segments (n=2). Hairpencil absent. Ovipositor broadly rounded.

*Measurements*. Male: forewing length 1.7–1.9 mm (n=2), wingspan: 3.8–4.3 mm. Female: forewing length 1.7–1.8 mm (n=3), wingspan 3.9–4.0 mm.

*Male genitalia* (Figs 70–72). Total length capsule 385–405µm (n=2), ventral plate very large and anteriorly rounded; tegumen rounded. Uncus distinctly bilobed, with setose lobes; gnathos with triangular pointed central element. Valva length 140–150 µm; transtilla well sclerotised, without sublateral processes. Phallus length 290 µm, flask shaped vesica with ca. 9 larger triangular cornuti; two elongate sclerotisations may represent the cathrema, no striate cathrema observed.

*Female genitalia* (Figs 74–76). Total length of bursa ca 660 µm. T8 with row of ca 8 setae on either side, partly on distinct sockets; no setose anal papillae. Anterior apophyses broadly rounded, posterior apophyses narrow, straight, longer than anterior ones. Vestibulum more strongly stained, with indistinct sclerotisation; ductus bursae not demarcated from corpus bursae, corpus asymmetric, curved; wall completely devoid of spines or pectinations. Ductus spermathecae slightly curved, only one incomplete convolution at vesicle.

##### Biology.

*Host plants* (Fig. 81). Melastomataceae: *Conostegia oerstediana* Ozadelpha Berg ex Triana and *Conostegia pitierri* Cogn., evergreen trees. Both species are distributed from Nicaragua to Panama, and recorded between 700 and 2400 m elevations in Costa Rica (Almeda 2007). *Conostegia oerstediana* is one of the widespread trees in the mid-elevation cloud forest of Costa Rica (K. Nishida, personal observation).

**Figures 80–87.**
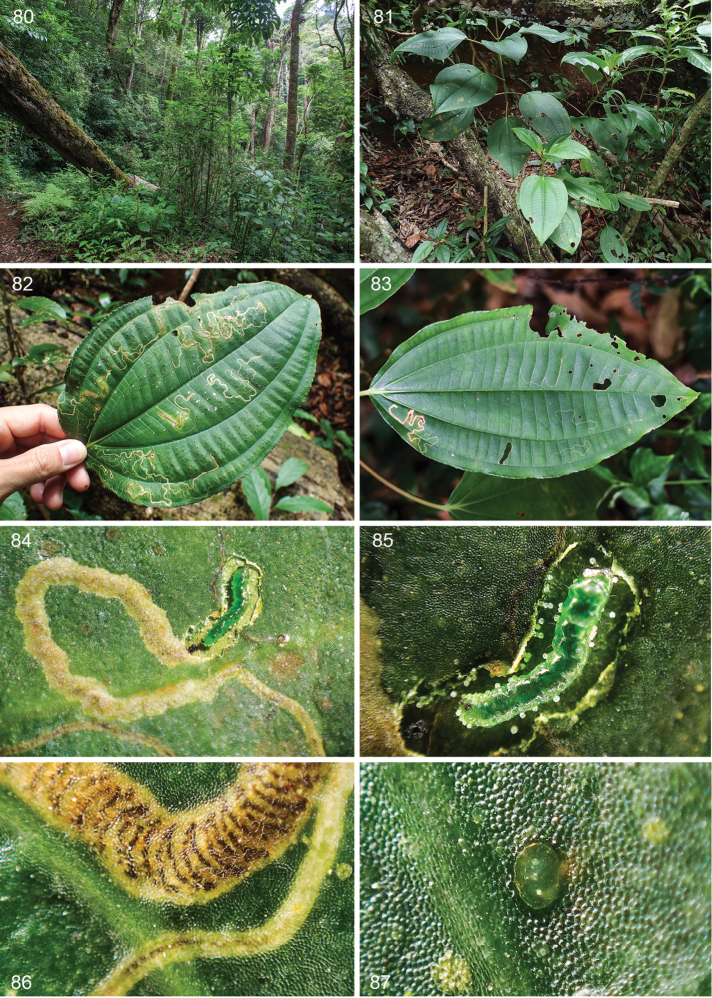
*Ozadelpha conostegiae*, habitat, host plant, *Conostegia oerstediana* and leafmines at Costa Rica, Estación Biológica Monteverde 1530 m. **80** Habitat, a cloud forest (lower mountain wet forest), 8 June 2016 **81** Treelets, infested with some leaf-mining larvae **82** Old leaf mines on host leaf, 24 March 2016 **83** Old leaf and young mines, 25 April 2016 **84, 85** Late instar larva in situ, upper epidermis removed to show larva, calcium oxalate crystals (druses) are not eaten and left behind, 18 May 2016 **86** Close-up of leaf mine, upper mine is of late instar larva, note the zigzag frass line, lower mine is of early instar, 7 June 2016 **87** Egg scale, dorsal view, attached to upperside of host leaf, 7 June 2016.

**Figures 88–94.**
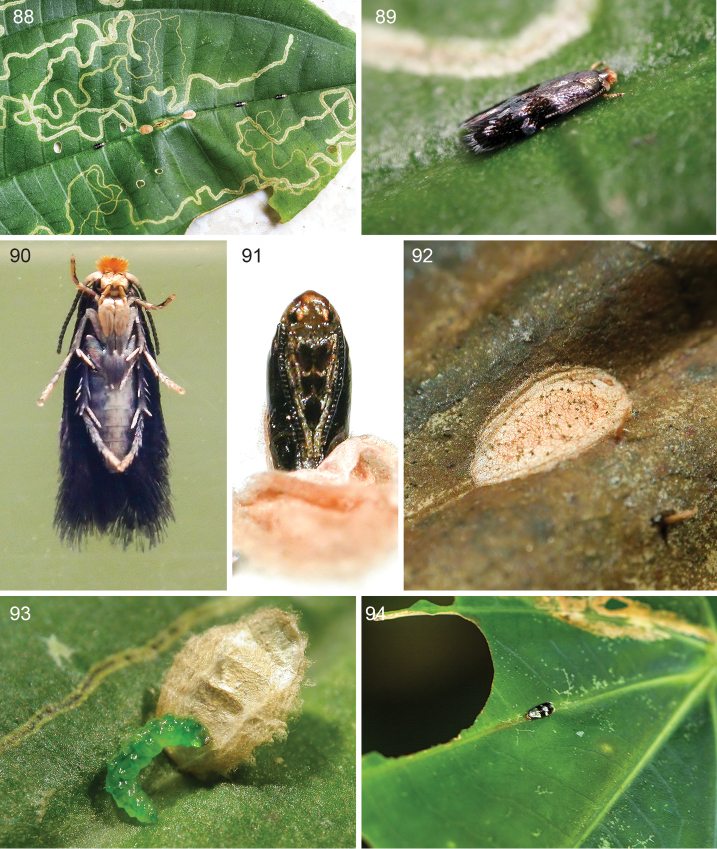
*Ozadelpha conostegiae*, life history details, Costa Rica, Estación Biológica Monteverde 1530 m. **88** Leaf mines, 2 cocoons and 3 adults on midrib on upperside host plant leaf, 29 March 2012 **89** Close up of one adult male **90** Adult female resting on plastic bag under rearing conditions, ventral view, 1 December 2013 **91** Pupa, ventral view, cocoon detached and removed, 7 June 2016 **92** Cocoon spun on upperside on secondary vein under captive conditions, 7 June 2016 **93** Cocoon-spinning mature larva, outer layer cocoon is removed to show larva, 19 June 2016 **94** *Ozadelpha* specimen EvN4680, female, resting on upper side leaf along one of the primary veins, 3 February 2012.

*Leafmines* (Figs 82, 83, 88). Narrow zigzag linear mine, pale brown in colour, on upperside leaf (n=42). Mature mine approximately 50 mm long (n=42). Some mines were found along leaf veins, i.e. mines were angular or square (n=7). The mines were mostly found on mature broad leaves of small treelets of less than a meter tall (n=ca 30). We recorded from a single mine up to 20 mines per leaf, with an average of 5 mines/leaf (n=15). Central portion of leaf mines filled with black frass, deposited in zigzag arcs (Fig. 86). Exit hole on underside of leaf at tip of mine, ellipsoid, 0.6–0.9 mm wide (n=5). Mines were found on leaves with a length of ca. 7–20 cm (mean 15.5) and width of ca. 4–13 cm (mean 9.75) (n=20).

*Egg* (Fig. 87). Laid singly, translucent with pale gold tint, oval, flat, egg case ca. 0.2 mm long, located contiguously to secondary leaf veins on upperside leaf (n=30). Some vacated egg cases were filled with frass (n=7).

*Larva* (Figs 84, 85, 93). Late to final instar larva translucent green, final instar larva 3.7 mm long (n=1).

*Cocoon* (Figs 92, 93). Oval shaped, flat, double chambered, outer cocoon 2.3–3.3 mm long and 1.3–2.0 mm wide, inner cocoon 1.6–2.0 mm long and 0.8–1.3 mm wide, pale brown to brown (n=4). Exit slit side of cocoon slightly thinner and paler than opposite end. Under rearing conditions inside plastic bags, the larvae pupated on host plant leaf, next to leaf veins or vein grooves either on upper or underside leaf (n=20) (Figs 88, 92). A species of a solitary koinobiont-endo parasitoid wasp was found inside the inner chamber of cocoon (n=2).

*Pupa* (Fig. 91). General appearance of mature pupa flat and dark brown, 1.5–1.7 mm long (n=2).

*Voltinism and habits*. Old and young leaf mines were seen all year round on treelets found along trails in forest understory at the Estación Biológica Monteverde. The larvae pupate away from the mine and host plant under natural conditions; however, it is unknown where the larvae spin their cocoons except for a single cocoon that was found on the underside of a mined leaf, but this was parasitized by a parasitoid wasp. Late stage to mature mines collected on 2.iii.2016 produced adults on 28–29.iii.2016. Mines collected on 21.v.2016 produced mature pupae (n=2) and mature pupae of a parasitoid wasp inside the moth cocoon (n=2) on 7.vi.2016. Mines collected on 25.v.2016 produced 6 cocoons between 2–6.vi.2016. Two eggs on a single leaf collected on 7.vi.2016 produced very early mines of ca. 1.2 mm and 8.6 mm long on 19.vi.2016. White, circular, pellet-like micro objects were found inside mines surrounding mining larvae (n=2) (Figs 84, 85). The pellets are calcium oxalate crystals (druses) according to R. Kriebel (personal communication). Thus the larvae appear to avoid feeding on druses.

*Parasitoid*. Eulophidae: Entedoninae: *Cornugon diabolos* Hansson (Hansson 2011), endoparasitoid, koinobiont of host larva which pupates inside host cocoon (n=2). This comprises a new host record for the genus *Cornugon* (Hansson 2011 and personal communication).

##### Distribution.

Costa Rica: Alajuela, Guanacaste and Puntarenas Provinces.

##### DNA barcode.

We failed to produce a DNA barcode from the DNA extracts, but we obtained sequences for 28S and COII from the holotype, RMNH.INS.24506. They will be made available through GENBANK with another paper in preparation.

##### Remarks.

*Ozadelpha conostegiae* represents the first published record of a Nepticulidae feeding on Melastomataceae. However, we also collected and reared unnamed *Acalyptris* species from the genus *Melastoma* from Australia: Queensland and Indonesia: Borneo. The genus *Conostegia* comprises 77 species of shrubs and trees in Central America, northern South America and the Carribean (Kriebel 2014).

##### Etymology.

The epithet *conostegiae* is a noun in genitive case, derived from the generic name of the host plant *Conostegia*.

##### Other material examined.

8♂, 6♀. **Costa Rica**: 1♂, Alajuela Province, Grecia, Reserva Forestal Grecia - Bosque del Niño, 10°.08'32.1"N, 084°.14'49.5"W, 1678 m, leafmines on *Conostegia oerstediana*, xii.2012 adult emergence, Kenji Nishida (RMNH); 1♂, 3♀, Guanacaste Province, Monteverde, Santa Elena, going towards Bosque Eterno de los Niños, San Gerardo Biological Station, 10°.21'25"N, 084°.47'35.1"W, 1500 m, leaf miner *Conostegia oerstediana*, emerged 23.iv.2012, Kenji Nishida, male abdomen missing, 3 female abdomens together in gelatin capsule, wing slide EvN4704, RMNH.INS.24704 (RMNH); 1♂, Puntarenas Province, Monteverde, Estación Biológia Monteverde, 10°19'06.9"N, 084°48'29.3"W, 1530 m, 2.iii.2012 leafmines, 31.iii.2012 adult emergence, *Conostegia oerstediana*, Kenji Nishida; 2♂, 2♀, same data, but 29.iii.2012 adult emergence, genitalia slide ♂ EvN4679, RMNH.INS.24679 (RMNH); 2♂, 1♀, same data, but late stage leaf mines collected 18–19.v.2016, adult emergence 18.vi.2016 (MZUCR); 1♂, Puntarenas Province, Monteverde, Estación Biológia Monteverde, 10°.19'13.95"N, 084°.48'20.83"W, 1600 m, 29.viii.2007 adult emergence, host plant: *Conostegia pitierri*, slide EvN 4844 [preparation of broken exuviae] (RMNH).

#### Ozadelpha

Taxon classificationAnimaliaLepidopteraNepticulidae

specimen EvN4680

##### Differential diagnosis.

Externally very similar to *Ozadelpha conostegiae*, but markedly larger (forewing length 2.8 mm against 1.8–2.0). Female genitalia recognised by longer anterior apophyses and very short bursa.

##### Female

(Figs 69, 94). Head: frontal tuft yellow orange, scape white; antenna broken. Collar indistinct, comprising lamellar scales, leaden. Thorax shining brass, forewing with basal leaden patch, a silver fascia at 2/3, narrowed in middle, at dorsum widened in both directions, silver scales along dorsum reaching basal patch. Hindwing scaling shining brown-grey. Abdomen dark fuscous, broadly rounded at tip.

*Measurements*. Female: forewing length 2.8 mm (n=1).

*Female genitalia* (Figs 77–79). T8 without setae. Anterior apophyses widened, ending in pointed process; posterior apophyses straight and narrow, longer than anterior ones. Total length bursa ca 340 µm, ductus bursae with one coil, corpus bursae asymmetric curved sac, devoid of any spines or pectinations. Ductus spermathecae without convolutions.

##### Biology.

*Host plants*. Unknown. The moth was found resting on the upperside of a *Conostegia oerstediana* (Melastomataceae) leaf along one of the main veins, thus this may well be the host.

*Voltinism and habits*. The moth was collected on March 2nd, 2012.

##### Distribution.

Costa Rica: Puntarenas Province.

##### Remarks.

We leave this species presently unnamed, as we only have a single female and no data on life history. There appear to be more closely related species of *Ozadelpha* feeding on *Conostegia* in Costa Rica, and we therefore rather await more material in order to be able to discriminate the various species better.

##### DNA barcode.

We barcoded the single specimen, currently the only DNA barcode we obtained from the genus *Ozadelpha*. This specimen was also sequenced for other genes and used in the molecular phylogeny (Doorenweerd et al. 2016). Sequences may be retrieved in BOLD and Genbank under voucher/sampled ID RMNH.INS.24680.

##### Material examined.

**Costa Rica**: 1♀, Puntarenas Province, Monteverde, Estación Biológica Monteverde, 1530 m, 10°19'06.9"N, 084°48'29.3"W, 2.iii.2012 resting on upperside of *Conostegia oerstediana* leaf along the vein, Kenji Nishida & Yuriko Demura; genitalia slide ♀ EvN4680, RMNH.INS.24680 (RMNH).

#### Neotrifurcula

Taxon classificationAnimaliaLepidopteraNepticulidae

van Nieukerken gen. n.

http://zoobank.org/E1E59003-CB86-45C0-B41A-E3E03BEF56FA

##### Type species.

*Neotrifurcula gielisorum* van Nieukerken sp. n. by present designation.

##### Diagnosis.

*Neotrifurcula* can be recognised by the hindwing venation with trifurcate Rs+M, and a very long and separate CuA in forewing, collar with hairscales, in the genitalia male phallus with a long curved flagellum-like appendix; female with reticulate signa and complex vaginal sclerite. *Glaucolepis* has a similar venation, but usually a velvet patch of special scales on hindwing and three pairs of anal tufts.

##### Description.

**Figure 95–96.**
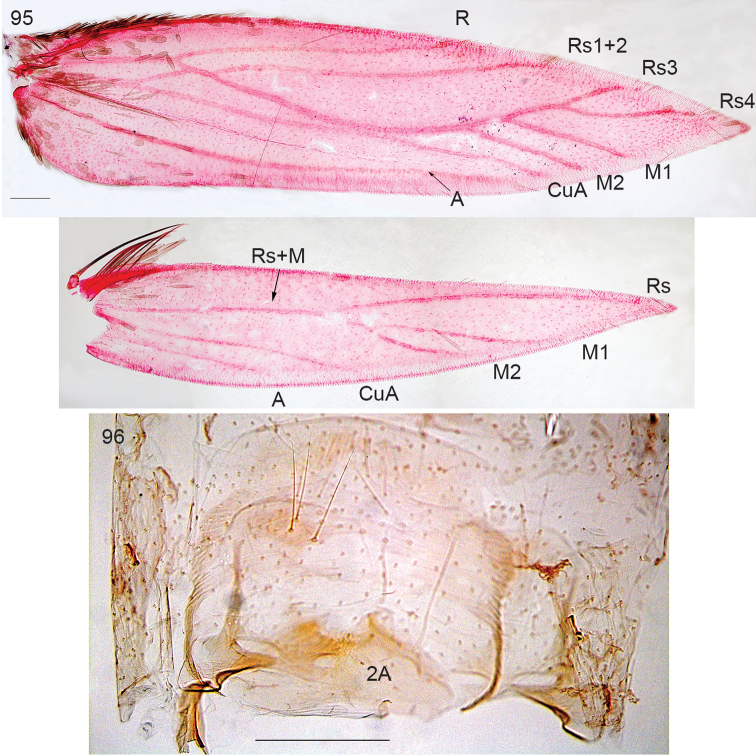
*Neotrifurcula gielisorum* gen. n., sp.n., male. **95** venation, veins labelled, slide EvN4703 **96** Abdominal segment 2, showing 2A, slide EvN4703. Scale bars: 200 µm.

**Figures 97–99.**
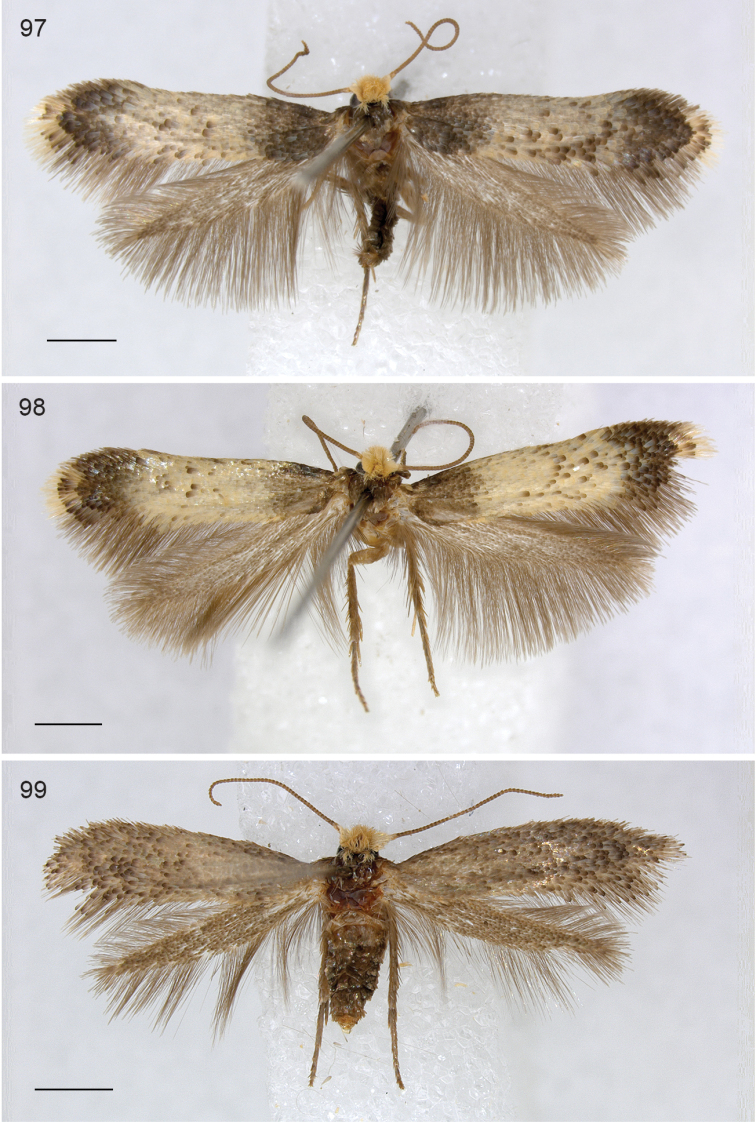
*Neotrifurcula* gen. n., adult habitus. **97** *Neotrifurcula gielisorum*, holotype male **98** *Neotrifurcula gielisorum*, male, RMNH.INS.23627 **99** *Neotrifurcula* specimen EvN4504, female, RMNH.INS.24504. Scale bars: 1 mm.

*Adult* (Figs 97–99). Medial to large nepticulid moths, forewing length 2.7–4.8 mm, largest over 10 mm wingspan. Head with collar comprising piliform scales; antenna with 42–58 segments in male (n=4), 47 in female (n=1). Forewing with distinct or less distinct fascia, sometimes metallic, no subdorsal retinaculum in male. Hindwing in male with costal bristles, no androconial scales observed. Venation (Fig. 95): very complete, with closed cell, R+Rs+M with 6 terminal branches: R, Rs1+2, Rs3, Rs4, M1 and M2, CuA separate and long, approaching Rs+M; A thickened; Hindwing broad, with 5 veins, Rs+M trifurculate: Rs, M1, M2. Abdomen: anterior part of sternum 2 with two lobes on posterior margin (Fig. 96). Tergum 8 with distinct anal tufts, tergum 3–7 with lateral groups of many setae and scales.

**Figures 100–103.**
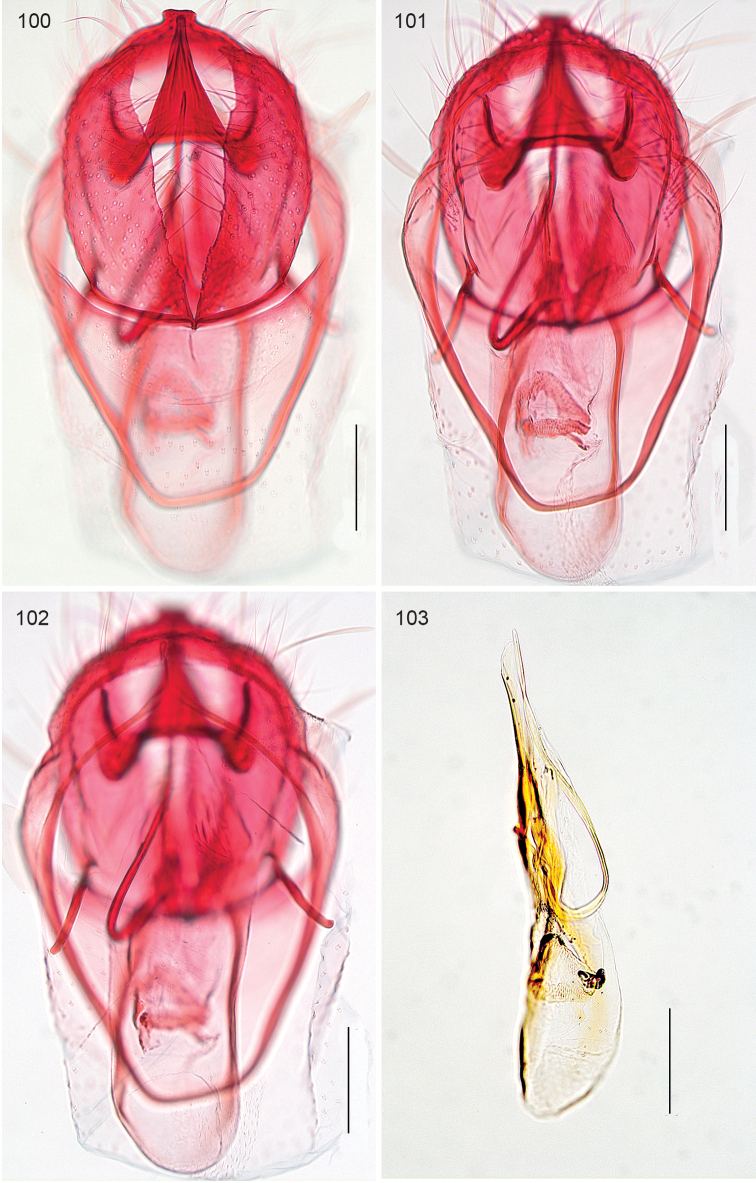
*Neotrifurcula gielisorum*, male genitalia. **100–102** holotype, various levels of focus **103** Phallus separate, lateral view, slide EvN4703. Scale bars: 100 µm.

**Figure 104.**
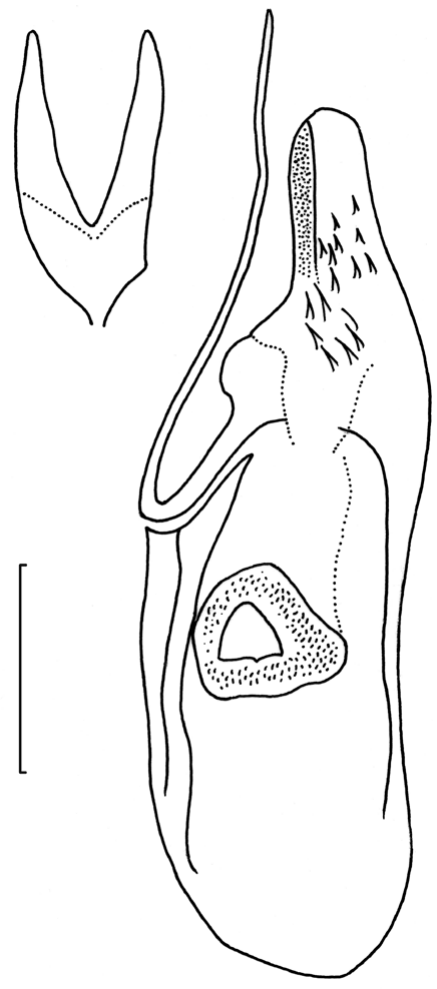
*Neotrifurcula gielisorum*, phallus and juxta (top left), ventral view, holotype. Scale: 100 µm.

*Male genitalia*. (Figs 100–104). Vinculum ring shaped, fused with tegumen; ventral plate expanded, not bilobed. Uncus Y shaped. Gnathos with large triangular central element. Valva elongate to triangular, transtilla without transverse bar, sublateral processes distinct. Juxta V-shaped. Phallus long, gradually tapering caudally; a peculiar long curved process at left side; vesica with small group of several cornuti.

*Female genitalia* (Figs 107–110). T9 a pair of setose anal papillae; T8 rounded, with a few setae. Anterior apophyses, slightly longer than posterior ones. Vestibulum with sclerotisation; corpus bursae with a pair of reticulate signa. Ductus spermathecae with 3.5 convolutions.

##### Biology.

Hostplant and immature stages unknown. Adults collected in *Nothofagus* forests from November to January.

##### Distribution.

Chile and Argentina, southern parts at low and medial altitudes.

##### Composition.

Next to the type species, we include two unnamed species, for one of which we only have one female that was sequenced, another one only one worn male on loan from Copenhagen (ZMUC). We find the DNA barcode distance too large to include the female in *Neotrifurcula gielisorum* and the male is much smaller and shows some differences in the genitalia. According to Jonas Rimantas Stonis (personal communication) there is a group of several closely related species of *Neotrifurcula* in Patagonia, estimated to comprise at least five species.

##### Etymology.

*Neotrifurcula*, a noun, a combination of the prefix *neo*-, new, here derived from Neotropics, and the Latin noun *Trifurcula* (= a three-pronged fork), another nepticulid genus with a 3-forked Rs+M in the hindwing. The name is to be treated as feminine.

##### Remarks.

The anterior sclerite of sternum 2 (S2A) has anterior apodemes similar to *Bohemannia* Stainton, 1859. The venation also has several similarities to *Bohemannia*, although the latter seems more reduced by the fusing of CuA with Rs+M and the reduction of the closed cell. This supports the possible sistergroup relationship to *Bohemannia* or *Bohemannia* + *Hesperolyra* that we found in our molecular analyses (Doorenweerd et al. 2016). Overall the species of *Neotrifurcula* resemble *Glaucolepis* in several ways: the venation is almost identical, the transverse bar of transtilla is absent, but *Neotrifurcula* does not have the male “velvet patch”, the phallotrema spines. The flagellum-like appendix of the phallus is a remarkable character of as yet unknown function. This character requires further detailed morphological study. It is likely an apomorphy for the genus.

#### Neotrifurcula gielisorum

Taxon classificationAnimaliaLepidopteraNepticulidae

van Nieukerken sp. n.

http://zoobank.org/A2E86BAF-5CB2-41A7-8F54-DDB5CF7D1BCA

##### Holotype male.

**Chile**, Ñuble, Bio-Bio (VIII), 2 km N Las Trancas, 70 km E Chilan, 1400 m, 36.54S-71.28W, 6.i.2001, C. Gielis & H. W. van der Wolf, sta 53, genitalia slide EvN4503, RMNH.INS.24503 (RMNH).

##### Differential diagnosis.

One of the largest nepticulids with a wingspan of almost 10 mm. Recognised by very broad cream fascia, male genitalia characteristic by flagellum-like appendix on phallus, but several closely related, but smaller species have very similar genitalia.

##### Description.

*Male* (Figs 97, 98). Head with frontal tuft pale yellow ochreous, collar similar, comprising hairscales; scale and pedicel similar, flagellum grey-brown. Antenna with 54–58 segments (n=3). Thorax fuscous, forewing fuscous with a very wide, irregular, pale cream medial fascia of ca. half wing length, with scattered fuscous scales; distally a double cilia line, separated by a cream patch. Hindwing broad, brown, costal bristles present, no androconials.

*Female*. Unknown.

*Measurements*. Male: forewing length 4.0–4.8 mm (n=3), wingspan: 8.7–10.1 mm.

*Male genitalia* (Figs 100–104, 111–114). Capsule length 460–480 µm. Tegumen fused with vinculum, ring-shaped; vinculum extended anteriorly. Uncus with medial truncate process, slightly dilated apically. Gnathos with large triangular central element. Valva length ca 265–270 µm, narrow, elongate, inner margin slightly sinuous, tip triangular. Transtilla without transverse bar, sublateral processes distinct. Juxta V-shaped, joining valvae and phallus. Phallus length 450–490 µm, gradually tapering caudally; a long curved process left side, first curved anteriorly, then making a 180 degrees turn to the dorsal side and ending posteriorly, close to phallotrema; vesica with small group of cornuti.

##### Biology.

*Host plants*. Unknown.

*Voltinism and habits*. The moth was collected from mid-December to mid-January.

##### Distribution.

**Figures 105–106.**
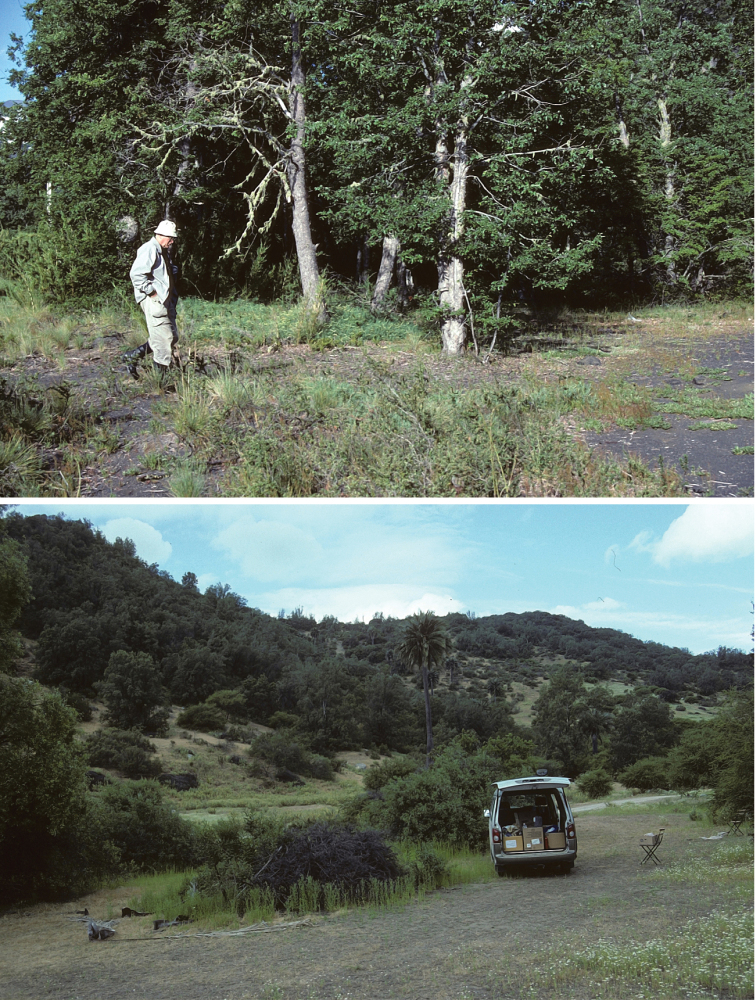
*Neotrifurcula* sp., habitats in Chile **105** (top), Type locality *Neotrifurcula gielisorum*, Nuble, Bio-Bio, 2 km N Las Trancas, 1400 m, 6 January 2001, edge of *Nothofagus* forest **106** (bottom) Locality for *Neotrifurcula* specimen EvN4504 Valparaiso, Parque Nat. La Campana, 450 m, *Nothofagus* forest near brooklet, photo 8 November 2000, specimen collected here 19 February 2001.

Chile: Curoco, Ñuble and Valparaiso. In both localities with dense forest of large *Nothofagus* trees at middle altitudes (1100–1500 m) (Fig. 105).

##### DNA barcode.

We barcoded two specimens, including the holotype, both in BIN BOLD ACG8607. One specimen was also sequenced for other genes and used in the molecular phylogeny (Doorenweerd et al. 2016). Sequences may be retrieved in BOLD and Genbank under voucher/sample ID RMNH.INS.23527.

##### Etymology.

The specific name *gielisorum* is a noun in plural genitive, based on the family name Gielis, to honour Cees and Siska Gielis for their efforts not only to collect this species, but to explore and collect Microlepidoptera widely in South America, and to publish in particular about the plume moths, Pterophoridae.

##### Other material examined.

**Chile**: 1♂, Curico, Maule (VII), 60 km SE Molina, RN Radal Seite Tazas, 1100 m, 18–19.xii.2000, 35.28S-71.00W, C. Gielis & FK Gielis, sta. 45, genitalia + wing slide EvN4703 (RMNH); 1♂, Nuble, Bio-Bio (VIII), 2 km N Las Trancas, 70 km E Chilan, 1400 m, 36.54S-71.28W, 14.i.2001, C. Gielis & H. W. van der Wolf, sta 63, genitalia + wing slide EvN3527 (RMNH).

#### Neotrifurcula

Taxon classificationAnimaliaLepidopteraNepticulidae

specimen EvN4504

##### Differential diagnosis.

Externally similar to *Neotrifurcula gielisorum*, but no distinct fascia. Female genitalia characterised by ductus spermathecae with 3.5 convolutions, distinct reticulate signa and omega-shaped sclerotisation in vestibulum.

##### Description.

*Male*. Unknown.

*Female* (Fig. 99). Head with frontal tuft pale yellow ochreous, collar similar, comprising hairscales; scale and pedicel similar, flagellum grey-brown. Antenna with 47 segments. Thorax descaled, forewing somewhat worn, fuscous with pale cream basal patch of ca. half wing length, with scattered fuscous scales and some metallic silver scales. Hindwing broad, brown, costal bristles present. Ovipositor rounded.

*Measurements*. Female: forewing length 3.8mm, wingspan 8.2 mm.

**Figures 107–110.**
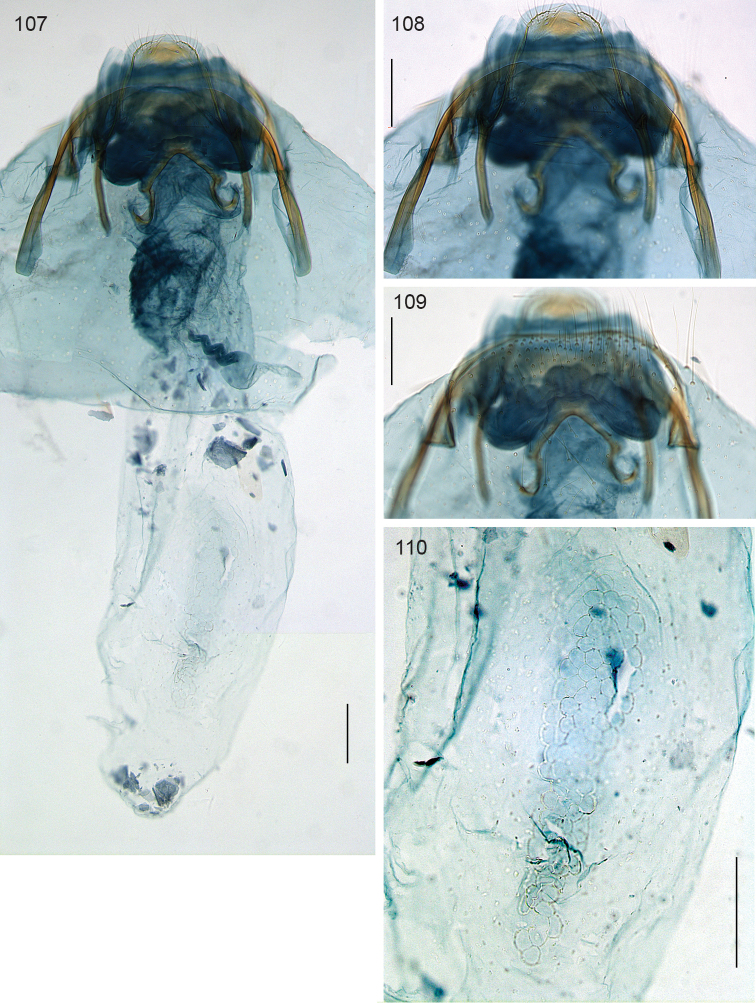
*Neotrifurcula* specimen EvN4504, female genitalia, slide EvN4504. Scale bars: 100 µm.

*Female genitalia* (Figs 107–110). T9 forming a pair of protruding anal papillae with 12 setae each; T8 rounded, with a few setae. Anterior apophyses anteriorly widened, slightly longer than posterior ones. Total bursa length 1160 µm long. Vestibulum with an omega shaped sclerotisation surrounding entrance of ductus bursae (Fig. 109); a pair of reticulate signa of ca 340 µm long, maximum 5 cells wide (Fig. 110); wall of bursa devoid of pectinations. Ductus spermathecae with 3.5 convolutions.

##### Biology.

*Host plant*. Unknown.

*Voltinism and habits*. The moth was collected in February.

##### Distribution.

Chile: Valparaiso. Collected in open shrubby habitat, with shrubby species of *Nothofagus* (Fig. 106).

##### DNA barcode.

We obtained a full barcode of the specimen, with BIN BOLD:ACU6693, showing a distance to barcodes of *Neotrifurcula gielisorum* of ca 12%.

##### Remarks.

The huge barcode distance to *Neotrifurcula gielisorum* and the differences in the wing pattern show clearly the species status of this specimen. It could possibly be the female of the next species, but difference in size and number of antennal segments make this rather unlikely.

##### Material examined.

1♀, **Chile**: Valparaiso, Parque Nat. La Campana, 6 km E of Olmue, 450 m, 33.00S-71.03W, 19.ii.2001, R.T.A. Schouten & H. W. van der Wolf, station 90, Nothofagus forest near brooklet, genitalia slide EvN4504 (RMNH).

#### Neotrifurcula

Taxon classificationAnimaliaLepidopteraNepticulidae

specimen RH2

##### Differential diagnosis.

The moth is markedly smaller than the previous two species, but too worn to see external diagnostics. Male genitalia similar to *Neotrifurcula gielisorum*, but smaller, valva more triangular and more distinct and cornuti larger.

##### Description.

*Male* Antenna with 42 segments, forewing length 2.7 mm. Head pale, forewings and thorax brown, very worn, pattern not visible.

*Female* unknown.

*Measurements*. Male: forewing length 2.7mm.

**Figures 111–114.**
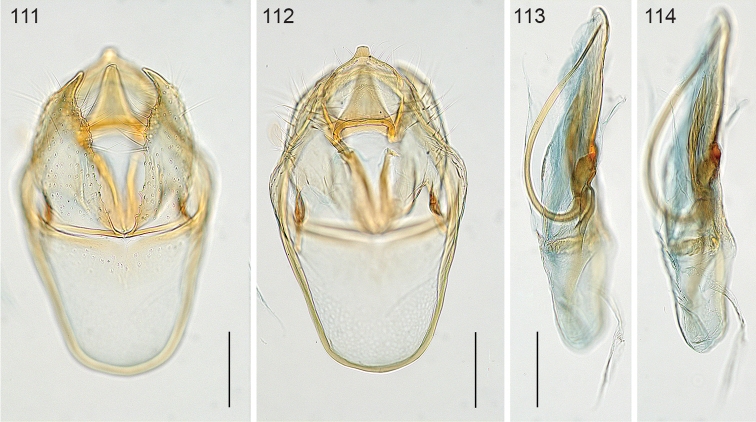
*Neotrifurcula* specimen RH2. Male genitalia, slide RH SA2. Scale bars: 100 µm.

*Male genitalia* (Figs 111–114). Capsule length 430 µm. Tegumen fused with vinculum, ring-shaped; vinculum extended anteriorly. Uncus with medial truncate process. Gnathos with large triangular central element. Valva length ca 215 µm, approximately triangular, tip pointed. Transtilla without transverse bar, sublateral processes distinct but rather short. Juxta V-shaped, joining valvae and phallus. Phallus length 450 µm, gradually tapering caudally; a long curved process left side, first curved anteriorly, then making a 180 degrees turn to the dorsal side and ending posteriorly, close to phallotrema; vesica with group of distinct cornuti.

##### Biology.

*Host plant*. Unknown.

*Voltinism and habits*. The moth was collected in November, around the conifer *Podocarpus salignus* D. Don (on the label incorrectly cited as *saligna*). It is unlikely that this represents the host.

##### Distribution.

Chile: Valdivia.

##### Remarks.

The moth had been studied by Robert Hoare for his thesis, and is part of a larger series that is under study with the group of J.R. Stonis (Vilnius) and probably will be described by them.

##### Material examined.

1♂, **Chile**, Valdivia, 20 km S Valdivia, Rincon de la Piedra, 15.xi.1981, Nielsen & Karsholt, station 15, caught around *Podocarpus saligna*, Genitalia and wing slide R. Hoare South America 2 (ZMUC).

#### Hesperolyra

Taxon classificationAnimaliaLepidopteraNepticulidae

van Nieukerken gen. n.

http://zoobank.org/BDA8170A-00CA-4613-8944-8DDE631A01D2

Fomoria molybditis group Puplesis et al. 2002b: 66.

##### Type species.

*Fomoria diskusi* Puplesis & Robinson, 2000: 43, by present designation.

##### Diagnosis.

There are no obvious external characters apart from the venation: broad forewing with a straight main vein with 4 branches, CuA absent, rather resembling *Acalyptris*, but no vestigial closed cell present and wings broader. The most obvious characters are in the male genitalia: bifurcate pseuduncus, deeply divided valva and long extended lyre-shaped transtilla. Female genitalia without reticulate signa, small bursa.

##### Description.

**Figures 115–116.**
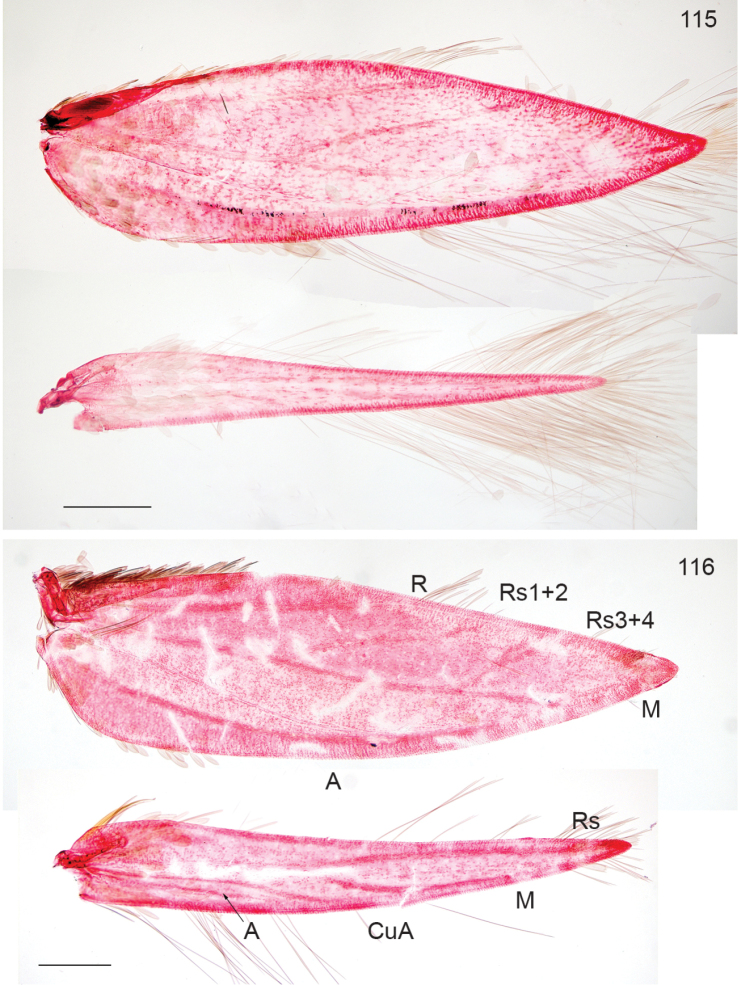
*Hesperolyra* gen. n., venation. **115** *Hesperolyra diskusi*, male paratype, slide EvN4501 **116** *Hesperolyra saopaulensis*, female, veins labelled, slide EvN4505. Scale bars: 200 µm.

**Figures 117–120.**
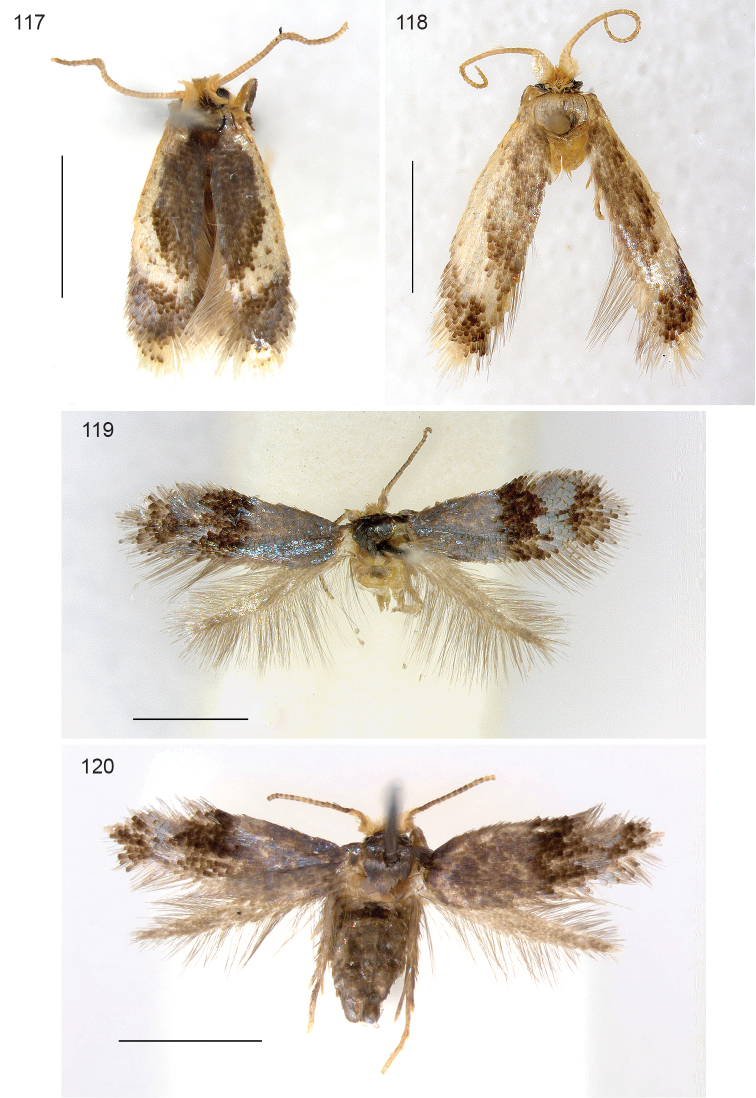
*Hesperolyra* gen. n., adult habitus. **117** *Hesperolyra diskusi*, male paratype, RMNH.INS.24501 **118** *Hesperolyra diskusi*, female paratype, BMNH(E)1625477 **119** *Hesperolyra saopaulensis*, female holotype **120** *Hesperolyra saopaulensis*, female RMNH.INS.24505. Scale bars: 1 mm.

**Figures 121–127.**
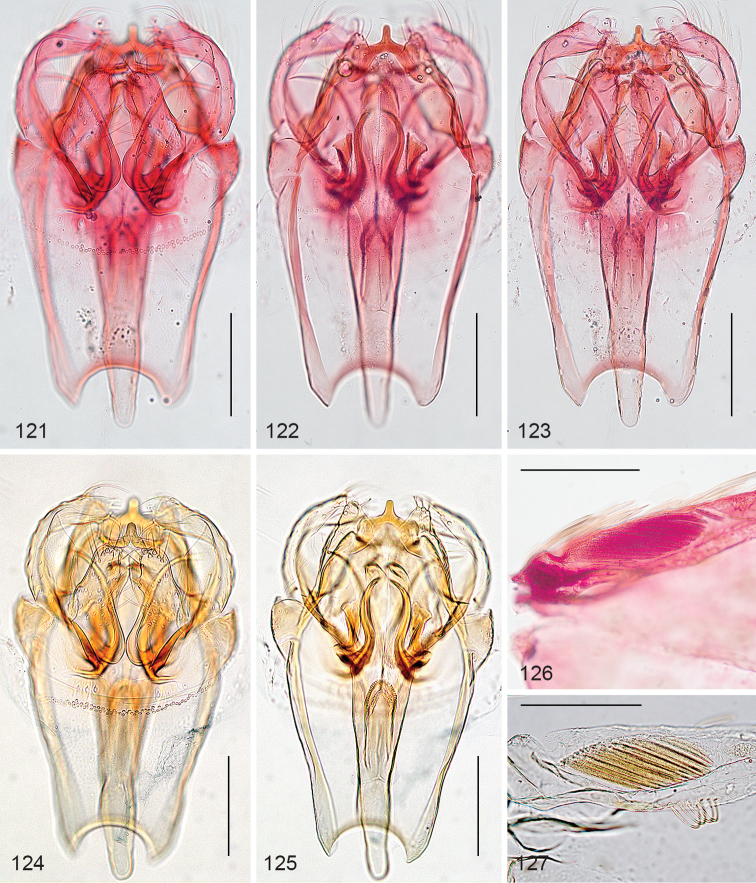
*Hesperolyra diskusi*, paratypes, male genitalia and forewing fold. **121–123** slide EvN4501 **124–125** slide BM29130 **126–127** Forewing fold and costal retinaculum, showing special scales hidden under fold, stained slide EvN4501, unstained, slide BM28847. Scale bars: 100 µm.

*Adult* (Figs 117–121). Small to large nepticulid moths, forewing length 1.8–4.0 mm. Head with collar comprising piliform scales. Antenna with 24–40 segments in male, 23–33 in female. Forewing variously patterned, no subdorsal retinaculum in male. Hindwing in male without costal bristles, no androconial scales observed. Forewing fold with group of hidden androconials in *Hesperolyra diskusi* (Figs 126, 127). Venation (Figs 115, 116): simplified, forewing rather broad, R separate from base, a straight main Rs+M, without closed cell, with 3 branches: Rs1+2, Rs3+4 and M; CuA absent (or fused), A thickened; Hindwing broad, with 3 or 4 visible veins, a bifurcate Rs+M, CuA and A. Tergum 8 with anal tufts.

*Male genitalia*. (Figs 121–125). Vinculum ring shaped, fused with tegumen; ventral plate expanded, very long, slightly excavated. Tegumen forming a bilobed pseuduncus. Uncus Y shaped. Gnathos with small central element. Valva complex, usually deeply divided, elongate to triangular, transtilla with enlarged transverse bar, forming a lyre-shaped anterior extension, comprising distinct but small sublateral processes. Various elaborate long spines either as part of valva or as ventral process (juxta). Phallus long, vesica often with many cornuti.

*Female genitalia* (Figs 129–136). T9 a pair of setose anal papillae; T8 broadly rounded, with some setae. Anterior apophyses slightly shorter than posterior ones. Vestibulum without sclerotisation; corpus bursae without signa, rather flimsy, a small folded accessory sac leading to a coiled ductus spermathecae with 2–7 convolutions.

*Pupa* (Figs 140–141). Frons in eclosion separated from scape. Abdominal tergites 2–8 with each ca 4 rows of spines.

##### Biology.

So far the host plant is only known for one species, *Hesperolyra saopaulensis*: Myrtaceae, on which the larva makes normal gallery mines (Figs 137–138).

##### Distribution.

Only known from the Neotropics: Belize, Brazil, Colombia and Ecuador.

##### Composition.

We recombine here the species previously placed in the *Fomoria molybditis* group: *Hesperolyra diskusi* (Puplesis & Robinson, 2000), comb. n., *Hesperolyra molybditis* (Zeller, 1877), comb. n., *Hesperolyra repanda* (Puplesis & Diškus, 2002), comb. n. and describe one new species, *Hesperolyra saopaulensis* van Nieukerken sp. n. One unnamed species also belongs here: *Hesperolyra* species 29122 (Puplesis and Robinson 2000).

##### Etymology.

The name *Hesperolyra* is a combination of Hespero- from Hesperus (the evening star), referring to the occurrence in the western hemisphere, and lyra (lyre), referring to the lyre shaped transtilla, a common character for species where the male is known. The name is to be treated as feminine.

##### Remarks.

The present genus was recognised first in our molecular analysis by the new species *Hesperolyra saopaulensis*, that consistently grouped outside any known genus, often together with *Neotrifurcula*, but at a large distance. Since we did not have a male of *Hesperolyra saopaulensis*, a generic description seemed problematic, until we noticed similarity to the recently obtained DNA barcode of *Fomoria diskusi* and the unusual venation of both species, quite different from any other *Fomoria*. *Fomoria diskusi* was placed with some other species in the *Fomoria molybditis* group (Puplesis et al. 2002b), here treated as a synonym of *Hesperolyra*. Puplesis and Robinson (2000) placed *Hesperolyra molybditis* and *diskusi* in *Fomoria* on the basis of superficial similarity of male genitalia, even though the venation is markedly different, resembling more that of *Acalyptris*. The authors even stated that “the unusually reduced wing venation lends additional support.” The lack of apomorphies for *Fomoria* s. str. make assignment of any species to that genus difficult without molecular support, and even with eight genetic markers the support is not high (Doorenweerd et al. 2016). After careful comparison we are convinced that the species in this group and *Hesperolyra saopaulensis* are congeneric. We selected *Hesperolyra diskusi* as type species, because it is the only species for which males and females are known. By this action, *Fomoria tabulosa* Puplesis & Diškus, 2002 remains the only known Neotropical species of *Fomoria*. Whether this indeed belongs to the clade to which the type species of *Fomoria*, *Fomoria weaveri* (Stainton, 1855), belongs, remains unclear, the genitalia are rather atypical and the venation has not been studied.

#### Hesperolyra diskusi

Taxon classificationAnimaliaLepidopteraNepticulidae

(Puplesis & Robinson, 2000) comb. n.

Fomoria diskusi Puplesis & Robinson, 2000: 43. Holotype ♂: BELIZE: Cayo Distr., Chiquibul For. Res., Las Cuevas, 3–16.iv.1998, R. Puplesis & S. Hill, Genitalia slide BM28844 (BMNH). [examined]

##### Differential diagnosis.

*Hesperolyra diskusi* is easily recognisable by its striking pattern: the pale costal streak that turns into an outward oblique fascia at 2/3. In the male genitalia the characteristic long spines separate it from congeners.

##### Redescription.

*Male* (Fig. 117). Frontal tuft yellow to orange; scape shining yellowish white, antenna with 37–39 segments. Collar of hairscales similar to frontal tuft. Thorax and forewing fuscous; yellowish white pattern comprising a costal streak running from wing base to slightly over 1/2, then changing into an outwards oblique fascia running to 2/3 of dorsum, and becoming narrower towards dorsum; scales along costa stronger yellow; terminal cilia yellowish white beyond an irregular cilia line. Thorax anteriorly pale, posteriorly fuscous, in rest joining to the forewing pattern. Forewing under fold with a conspicuous row of special scales, only visible in descaled wings (Figs 126, 127).

*Female* (Fig. 118). Antenna with 33 segments. Otherwise as male.

*Measurements*. Male: forewing length 1.8–2.3 mm (n=2), wingspan 4.0–5.0 mm. Female: forewing length 2.1 mm (n=1), wingspan 4.6 mm.

**Figure 128.**
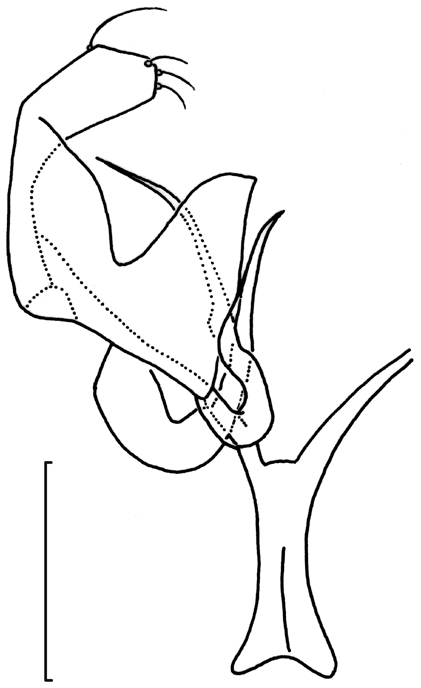
*Hesperolyra diskusi*, paratype, slide EvN4501, detail of left valva and lyre-shaped transtilla. Scale: 100 µm.

*Male genitalia* (Figs 121–125, 128) (n=3). Total length capsule ca 330–350 µm. Vinculum long, anteriorly with small excavation; tegumen in middle with excavation, resulting in bilobed pseuduncus; uncus inverted Y-shaped; gnathos almost similar in shape to uncus. Valva 165–190 µm, elaborate bilobed structure, with curved outer lobe and rather straight flat inner lobe, and three elaborate long and curved spines that attach to the anellus; transtilla without sublateral processes, transverse bar extending anteriorly in a lyre-shaped plate. Phallus ca 320–350 µm long, tapering anteriorly, posteriorly attached to the long valval spines, some of which may actually be carinal processes.

**Figures 129–131.**
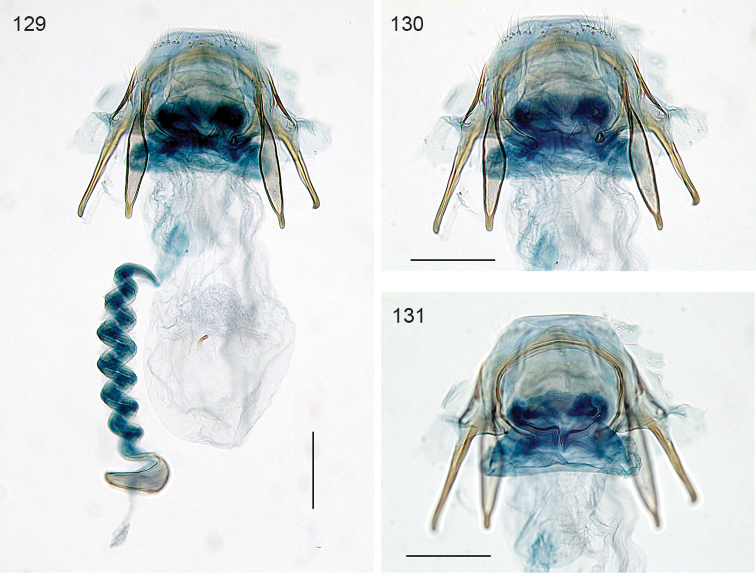
*Hesperolyra diskusi*, female genitalia, paratype, slide BM28846. Scale bars: 100 µm.

*Female genitalia* (Figs 129–131). T9 forming broad pair of anal papillae with each ca 15 setae. T8 with rather square posterior margin, some setae on either side. Anterior apophyses narrow, posterior apophyses slightly widened, of about same length. Total length of bursa 460 µm. Bursa without signa or other ornamentation. Ductus spermathecae distinct and longer than corpus bursae, with 7 convolutions and ending in wide and pointed vesicle.

##### Biology.

Host plant unknown. Collected at light in April, during the dry season in secondary forest (Puplesis and Robinson 2000).

##### Distribution.

Only known from Belize (*ca.* N16.732, W88.988).

##### DNA barcode.

We barcoded one paratype (RMNH.INS.24501), BOLD:ACY4502.

##### Other material examined.

4♂, 1♀, paratypes: **Belize**: Cayo District, Chiquibul Forest Reserve, Las Cuevas, 3–16.iv.1998, R. Puplesis & S. Hill, Genitalia slides ♂ EvN4501 (+ wings), BM28845, BM29130, ♀ BM28846, wing slide BM28847, resp. RMNH.INS.24501 (RMNH), BMNH(E)1625433, 1625439, 1625481, 1625477 (♀) (BMNH).

#### Hesperolyra saopaulensis

Taxon classificationAnimaliaLepidopteraNepticulidae

van Nieukerken sp. n.

http://zoobank.org/70F29918-65D6-457C-A635-400809BCEA3A

##### Holotype female.

**Brazil**, São Paulo, Garulhos, park near airport terminal, 19.viii.2000, EvN no 2000133 [rearing number], leafmines on unidentified Myrtaceae, E.J. van Nieukerken, adult emergence 11–14.ix.2000, RMNH.INS.23553, genitalia slide EvN3553, type number DZ 33.340 (DZUP).

##### Differential diagnosis.

Externally recognised by silver to leaden basal half of forewing and postmedial silvery fascia, with cilia line present and slightly edged scape. Female genitalia simple, without signa.

##### Description.

*Male*. Unknown.

*Female* (Figs 119, 120). Frontal tuft pale yellow; scape white, slightly edged with grey, antenna with 23 segments (n=1). Collar of hairscales similar to frontal tuft. Thorax and basal half of forewing shining leaden to silver; followed by a slightly postmedial fuscous fascia, then a silver fascia at 2/3 and a fuscous wingtip; terminal cilia silvery white beyond cilia line. Hindwing pale. Abdomen with blunt tip.

*Measurements*. Female: forewing length 1.9–2.1 mm (2.0 ± 0.1, 14), wingspan 4.0–4.6 mm.

**Figures 132–136.**
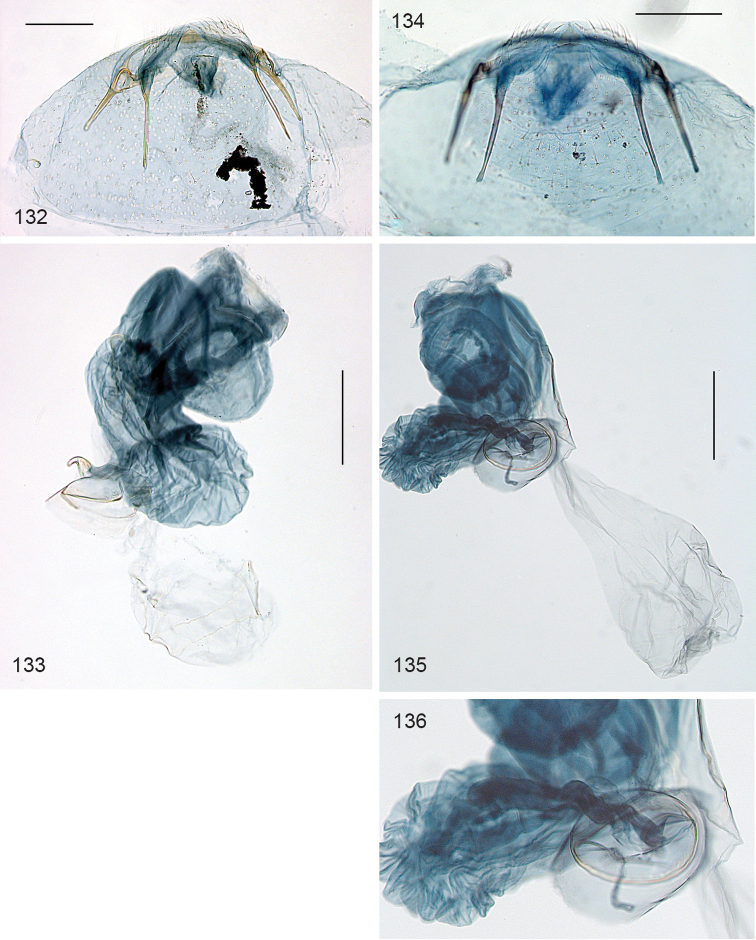
*Hesperolyra saopaulensis*, female genitalia. **132–133** Holotype, slide EvN3553 **134–136** Slide EvN4505 **136** enlargement of 135, detail of ductus spermathecae and vesicle. Scale bars: 100 µm.

*Female genitalia* (Figs 132–136). T9 forming broad pair of anal papillae with each ca 16–17 setae. T8 with truncate posterior margin, some setae on either side. Anterior and posterior apophyses narrow, posterior apophyses slightly longer. Total length of bursa 450–500 µm. Bursa without signa or other ornamentation; vestibulum folded and more sclerotised. Ductus spermathecae distinct, but short, with 2–3 indistinct convolutions and ending in wide and pointed vesicle.

##### Biology.

*Host plants*. Myrtaceae: unidentified tree (probably an *Eugenia* or *Myrcia* sp.).

**Figures 137–141.**
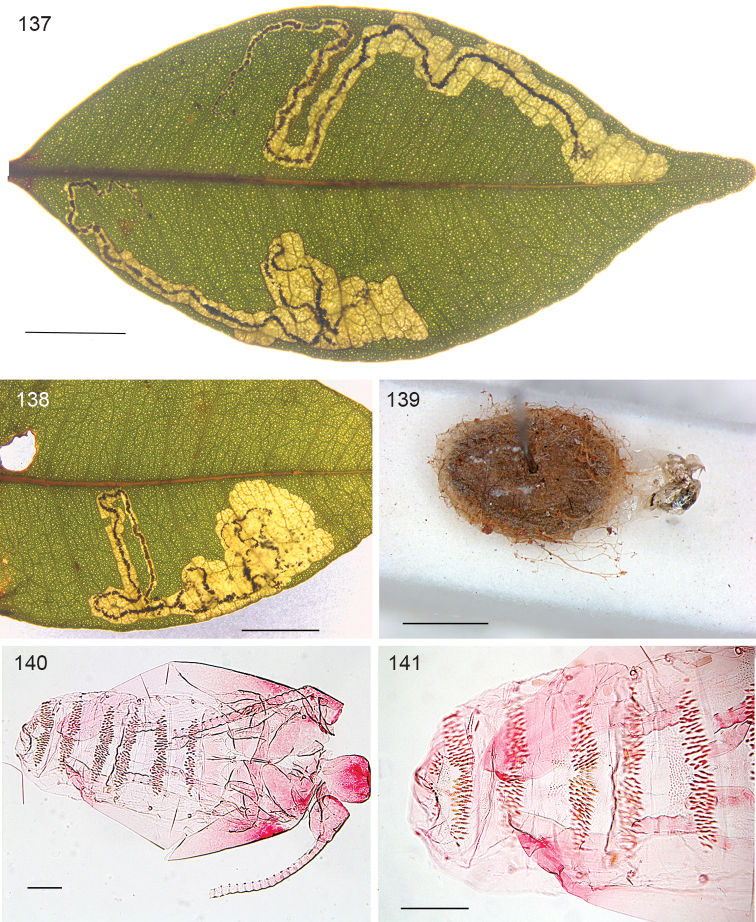
*Hesperolyra saopaulensis*, biology and immatures. **137–138** leafmines on unidentified Myrtaceae, sample EvN2000133K from which both adults were reared **139–141** Holotype cocoon and pupal exuviae, slide EvN3553. Scales: 5 mm (leaf mines), 1 mm (cocoon), 200 µm (pupa).

*Leafmines* (Figs 137, 138). The leafmine is a contorted gallery, with linear broken black frass throughout, sometimes forming a false blotch at the end. Larval emergence exit at leaf upperside.

*Egg*. The egg is deposited on leaf upperside, often on a lateral vein.

*Larva*. Not described.

*Cocoon* (Fig. 139). Brown, exuviae protruding.

*Voltinism and habits*. Larvae found in August, moths emerged in September.

##### Distribution.

Brazil: São Paulo.

##### DNA barcode.

We barcoded both specimens, resulting in BIN: BOLD:ACG9057. The Holotype was also sequenced for other genes and used in the molecular phylogeny (Doorenweerd et al. 2016). Sequences may be retrieved in BOLD and Genbank under voucher/sample ID RMNH.INS.23553.

##### Remarks.

Even though we have no males, we decided to name this species, of which we know the biology, several genes, and which is characteristic both externally and in female genitalia. Furthermore it is the species that was decisive in erecting the new genus *Hesperolyra*. Collecting new material should be easy, in the small park forest in front of the entrance of Garulhos airport.

##### Etymology.

Saopaulensis: an adjective, derived from the province and city name São Paulo and the suffix –*ensis*, indicating geographical origin.

##### Other material examined.

1♀, leafmines, **Brazil**, São Paulo, Garulhos, park near airport terminal, 19.viii.2000, EvN no 2000133 [rearing number], leafmines on unidentified Myrtaceae, E.J. van Nieukerken, adult emergence 11–14.ix.2000, genitalia slide Ev4505, RMNH.INS.24505 (RMNH).

### *Acalyptris* Meyrick, 1921

#### Acalyptris janzeni

Taxon classificationAnimaliaLepidopteraNepticulidae

van Nieukerken & Nishida sp. n.

http://zoobank.org/06E144D5-A691-4923-A9EF-24274CC65610

##### Holotype male.

**Costa Rica**, Guanacaste Province, ACG Santa Rosa Station, 10°50'22.30"N, 085°37'6.43"W, 293 m, 22.vi.2003, light sheet, Kenji Nishida, Genitalia slide EvN3673, RMNH.INS.23673 (RMNH).

##### Differential diagnosis.

Externally a dull species, not distinguishable from others without obvious colour pattern. The male genitalia are characterised by the bifid pseuduncus, lateral support rods and several large curved carinal spines.

##### Description.

**Figures 142–148.**
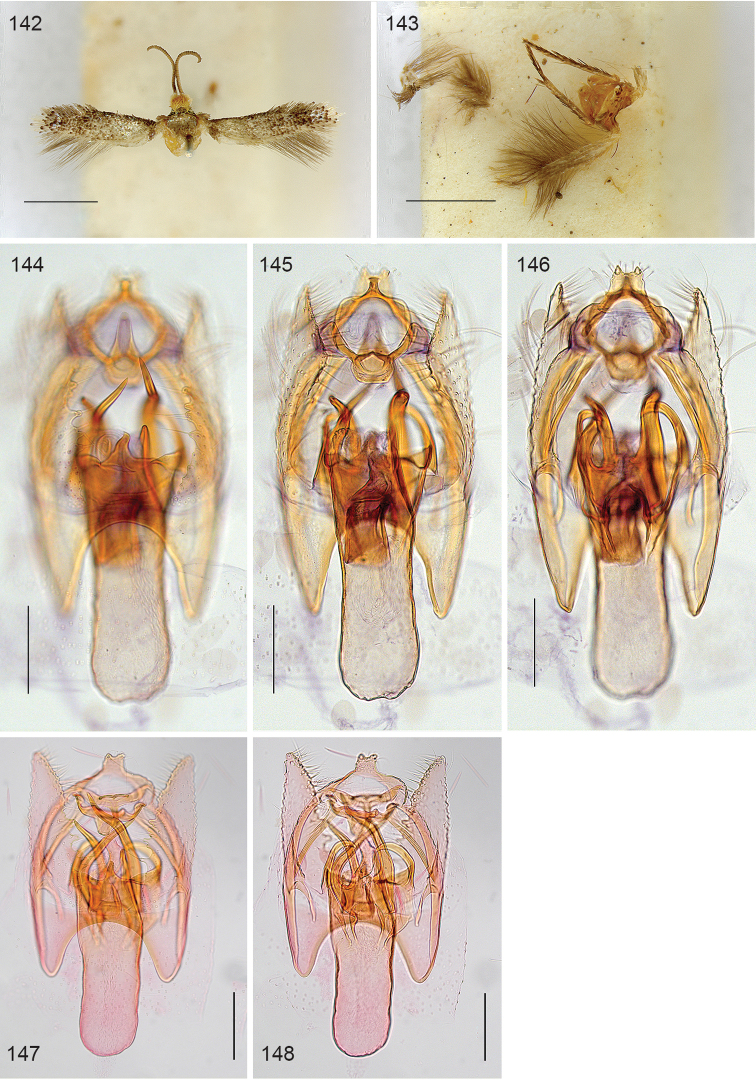
*Acalyptris janzeni*. **142–133** Holotype, metathorax and hindwings broken off and glued to the polyporus strip, scales 1 mm **144–146** Holotype, male genitalia. Slide EvN3673 **147–148** Male genitalia, slide JCK8232. Scales: 1 mm (**142–143**), 100 µm (rest).

*Male* (Figs 142, 143). Head: frontal tuft and collar pale yellow, scape and pedicel white, flagellum brown, antenna with 35–40 segments (n=4). Thorax and forewing irrorate yellowish white with brown, caused by dark tipped scales; an indistinct cilia line, terminal fringe white. Hindwing grey brown, no special scales; costal bristles present.

*Female*. Unknown.

*Measurements*. Male: forewing length 1.7 mm, wingspan: 4.2 mm.

**Figure 149.**
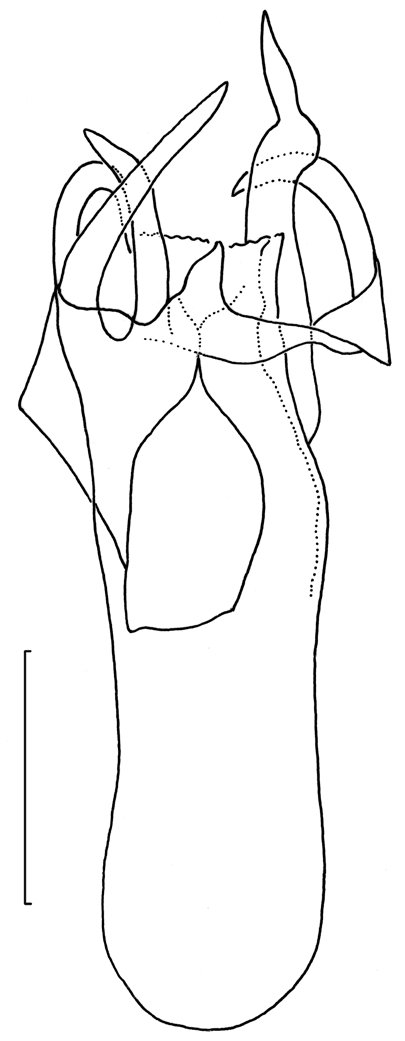
*Acalyptris janzeni*, phallus, holotype. Scale: 100 µm.

*Male genitalia* (Figs 144–149). Capsule length 250–280 µm. Vinculum ventral plate deeply bilobed; a pair of lateral support rods running from valval attachment to gnathos. Tegumen forming a bilobed pseuduncus. Uncus inverted Y-shaped, with lateral arms expanded, and central process distally widened. Gnathos with narrow triangular central element. Valva length ca 250–255 µm, narrow, with prominent setal sockets along inner margin, tip straight and pointed; sublateral processes distinct, transverse bar of transtilla absent. Phallus length ca 300 µm, carinal processes excluded; phallus wall ventrally with finger-shaped medial process; in total 5 long and curved carinal processes, the dorsal pair curving almost 180 degrees; vesica with indistinct plate-like sclerotisation, no cornuti observed.

##### Biology.

*Host plant*. Unknown.

*Voltinism and habits*. Adults found from late June to early August and again November to mid-March. Collected at light and in malaise traps.

##### Distribution.

Costa Rica: Guanacaste Province, Área de Conservación Guanacaste. Coordinates type locality: N10.83400, W85.61200. It is a sub-humid to humid tropical dry (deciduous) forest with five to six months of dry season (Herrera Soto and Gómez Pignataro 1993).

##### DNA barcode.

We have DNA barcodes of all ten known specimens, the holotype differs 2.3% from the other specimens.

##### Remarks.

The combination of the venation, absence of transverse bar of transtilla and presence of lateral support rods makes this a typical New World *Acalyptris* species, probably belonging to the *Acalyptris scirpi* group. The DNA barcode does not place it close to any *Acalyptris* species of which the barcode is known. Morphologically there are similarities to several species described from Belize: *Acalyptris bifidus* Puplesis & Robinson, 2000 and *Acalyptris unicornis* Puplesis & Robinson, 2000. It is remarkable that the holotype barcode has a 2.3% distance to the Malaise trapped specimens, found in almost the same locality. More material is needed to see whether this is a case of cryptic species, or a unusual high variation in the population.

##### Etymology.

Janzeni: a noun in genitive case, based on the family name Janzen, to honour Daniel H. Janzen, collector of part of the material, for his long time dedication to study the tropical Lepidoptera fauna of the Guanacaste Conservation area in great detail, both ecologically and taxonomically, and his enthusiastic support of DNA barcoding (eg. Janzen et al. 2009).

##### Other material examined.

6♂ [DNA barcoded ethanol material]: **Costa Rica**, Guanacaste, Área de Conservación Guanacaste, Sector Santa Rosa, Bosque San Emilio, Forest, Malaise trap, 300 m, 10.8438, -85.6138, Dan Janzen: 1♂, trap GMP#00624, 31.vii–6.viii.2012, BIOUG05419-H06, genitalia slide JCK8232; 1♂, trap GMP#01813, 6–13.xi.2012, BIOUG09432-B01; 2♂, trap GMP#01815, 20–27.xi.2012, BIOUG09436-B12, BIOUG09436-C06; 1♂, trap GMP#01817, 4–11.xii.2012, BIOUG09441-C02; 1♂, trap GMP#01825, 29.i–5.ii.2013, BIOUG10108-C05 (RMNH).

##### More data from BOLD

[specimens not examined, same BIN] 3 adults, same locality: trap GMP#01824, 22–29.i.2013, BIOUG18276-D11; trap GMP#01826, 5–12.ii.2013, BIOUG18337-A10; trap GMP#01830, 12–19.iii.2013, BIOUG18605-F04 (BIOUG).

## Supplementary Material

XML Treatment for Stigmella gallicola

XML Treatment for Stigmella schinivora

XML Treatment for Stigmella costaricensis

XML Treatment for Stigmella intronia

XML Treatment for Stigmella molinensis

XML Treatment for Ozadelpha

XML Treatment for Ozadelpha conostegiae

XML Treatment for Ozadelpha

XML Treatment for Neotrifurcula

XML Treatment for Neotrifurcula gielisorum

XML Treatment for Neotrifurcula

XML Treatment for Neotrifurcula

XML Treatment for Hesperolyra

XML Treatment for Hesperolyra diskusi

XML Treatment for Hesperolyra saopaulensis

XML Treatment for Acalyptris janzeni

## Supplementary materials

Supplementary material 1 Specimen data for Neotropical Nepticulidae with BOLD data Data type: Microsoft Excel (xls) Explanation note: The 87 records are all records of the new taxa described in this paper. Records are listed alphabetically by species, country, stateProvince, AdminDivision2, AdminDivision3, Locality. This dataset is made available under the Open Database License (http://opendatacommons.org/licenses/odbl/1.0/). The Open Database License (ODbL) is a license agreement intended to allow users to freely share, modify, and use this Dataset while maintaining this same freedom for others, provided that the original source and author(s) are credited.Erik J. van Nieukerken, Camiel Doorenweerd, Kenji Nishida, Chris Snyers
